# Genome-Wide Mouse Mutagenesis Reveals CD45-Mediated T Cell Function as Critical in Protective Immunity to HSV-1

**DOI:** 10.1371/journal.ppat.1003637

**Published:** 2013-09-12

**Authors:** Grégory Caignard, Gabriel A. Leiva-Torres, Michael Leney-Greene, Benoit Charbonneau, Anne Dumaine, Nassima Fodil-Cornu, Michal Pyzik, Pablo Cingolani, Jeremy Schwartzentruber, Jeremy Dupaul-Chicoine, Huaijian Guo, Maya Saleh, André Veillette, Marc Lathrop, Mathieu Blanchette, Jacek Majewski, Angela Pearson, Silvia M. Vidal

**Affiliations:** 1 Departments of Human Genetics and Medicine, McGill University, Montréal, Quebec, Canada; 2 School of Computer Science and McGill Centre for Bioinformatics, McGill University, Montréal, Quebec, Canada; 3 McGill University and Genome Québec Innovation Centre, Montréal, Quebec, Canada; 4 Departments of Biochemistry and Medicine, McGill University, Montréal, Quebec, Canada; 5 Laboratory of Molecular Oncology, Clinical Research Institute of Montréal, Montréal, Quebec, Canada; 6 INRS-Institut Armand-Frappier, Université du Québec, Laval, Quebec, Canada; Saint Louis University, United States of America

## Abstract

Herpes simplex encephalitis (HSE) is a lethal neurological disease resulting from infection with Herpes Simplex Virus 1 (HSV-1). Loss-of-function mutations in the *UNC93B1*, *TLR3*, *TRIF*, *TRAF3*, and *TBK1* genes have been associated with a human genetic predisposition to HSE, demonstrating the UNC93B-TLR3-type I IFN pathway as critical in protective immunity to HSV-1. However, the *TLR3*, *UNC93B1*, and *TRIF* mutations exhibit incomplete penetrance and represent only a minority of HSE cases, perhaps reflecting the effects of additional host genetic factors. In order to identify new host genes, proteins and signaling pathways involved in HSV-1 and HSE susceptibility, we have implemented the first genome-wide mutagenesis screen in an *in vivo* HSV-1 infectious model. One pedigree (named *P43*) segregated a susceptible trait with a fully penetrant phenotype. Genetic mapping and whole exome sequencing led to the identification of the causative nonsense mutation L3X in the *Receptor-type tyrosine-protein phosphatase C* gene (*Ptprc^L3X^*), which encodes for the tyrosine phosphatase CD45. Expression of *MCP1*, *IL-6*, *MMP3*, *MMP8*, and the *ICP4* viral gene were significantly increased in the brain stems of infected *Ptprc^L3X^* mice accounting for hyper-inflammation and pathological damages caused by viral replication. *Ptprc^L3X^* mutation drastically affects the early stages of thymocytes development but also the final stage of B cell maturation. Transfer of total splenocytes from heterozygous littermates into *Ptprc* ^L3X^ mice resulted in a complete HSV-1 protective effect. Furthermore, T cells were the only cell population to fully restore resistance to HSV-1 in the mutants, an effect that required both the CD4^+^ and CD8^+^ T cells and could be attributed to function of CD4^+^ T helper 1 (Th1) cells in CD8^+^ T cell recruitment to the site of infection. Altogether, these results revealed the CD45-mediated T cell function as potentially critical for infection and viral spread to the brain, and also for subsequent HSE development.

## Author Summary

Herpes simplex encephalitis (HSE) is a lethal neurological disease resulting from infection with Herpes Simplex Virus 1 (HSV-1). Previous studies have demonstrated a human genetic predisposition to HSE. However, the gene mutations that have been suggested as critical in protective immunity to HSV-1, exhibit incomplete penetrance and represent only a minority of HSE cases, perhaps reflecting the effects of additional host genetics factors. In order to identify new host genes involved in HSV-1 and HSE susceptibility, we have implemented the first genome-wide mutagenesis screen in an *in vivo* HSV-1 infectious model. Using this large-scale approach, we have identified a loss-of-function mutation in the *Receptor-type tyrosine-protein phosphatase C* (*Ptprc*) gene. Mice carrying this mutation were characterized by defects in thymic and B cell development. Following infection, these mutant mice exhibited hyper-inflammation in their brains stems caused by viral replication. Transfer of total lymphocytes from resistant into mutant mice resulted in a complete HSV-1 protective effect. Furthermore, T lymphocytes were the only cell population to fully restore resistance to HSV-1 in the mutants. These findings revealed the T cell function as potentially critical for infection and viral spread to the brain, as well as to subsequent HSE development.

## Introduction

Herpes simplex virus type 1 (HSV-1) is a large, enveloped virus of the *Herpesviridae* family. Its 152 kilobase (kb), double-stranded DNA genome encodes more than 80 polypeptides [1]. HSV-1 is among the most prevalent and successful human pathogens [2] and is typically transmitted through intimate contact and exchange of bodily fluids, such as saliva. This virus causes a life long infection, which consists of two distinct phases: an initial lytic stage, followed by a shift to latency once it reaches sensory neurons. Periodically, reactivation from latency occurs and is associated with numerous diseases, ranging from the common cold sore to ocular herpetic stromal keratitis, a leading cause of infectious blindness [3] [4]. Reactivation events as well as primary infections are also associated with herpes simplex encephalitis (HSE), a rare but life threatening consequence of infection of the central nervous system (CNS) [5]. In most of cases, the virus reactivates in the olfactory bulb or trigeminal ganglia, enters the brain via a retrograde axonal transport, replicates into the CNS, causing acute inflammation and significant pathological damages (review, see [6]). HSE is caused by direct lytic effects of the virus on neurons and glial cells, but mostly, by collateral damage, such as the disruption of the blood-brain barrier (BBB), due to the accompanying inflammatory reaction and leukocytes homing to the brain [7] [8] [9]. If left untreated, HSE is lethal in nearly 70% of cases; despite treatment, debilitating sequelae frequently develop [5] [10] [11]. In developed countries, it remains among the most common causes of viral encephalitis [12].

Since HSE was discovered in 1941, it has remained unclear why only a small proportion of otherwise healthy individuals exposed to HSV-1 develop the disease. In 2003, an autosomal recessive mutation in the *STAT1* gene was the first genetic etiology to HSE reported in HSV-1 seropositive patients [13]. More recently, loss-of-function mutations in the *UNC93B1*, *TLR3*, *TRIF*, *TRAF3*, and *TBK1* genes have also been associated with HSE in otherwise healthy children [14] [15] [16] [17] [18] [19], demonstrating the critical role of the UNC93B-TLR3-type I IFN pathway in the outcome of childhood HSE. Indeed, the fibroblasts of these patients displayed an impaired production of IFN-α, IFN-β and IFN-λ following TLR3 stimulation. These fibroblasts are also highly susceptible to infection with HSV-1 and this phenotype has recently been shown to recapitulate the impairment of TLR3-dependent HSV-1 control in UNC-93B deficient neurons and oligodendrocytes [20]. However, the *TLR3*, *UNC93B1*, and *TRIF* mutations exhibit incomplete clinical penetrance [21], perhaps reflecting the effects of additional environmental or host genetics factors. Furthermore, these mutations affect only a small proportion of children with HSE. Altogether, these observations suggest that predisposition to HSE may result from a set of diverse single gene defects and indicate the likely existence of other anti-HSE pathways.

Mouse models have also provided an efficient way to identify host factors that contribute to susceptibility or resistance to HSE. In 1975, Lopez and colleagues were the first to demonstrate the contribution of host genetics to HSE pathogenesis using different inbred strains of mice with varying resistance to HSV-1 [22]. Subsequently, HSE mouse models have employed several viral and mouse strains, knock-out mice, as well as various routes of infections. It has been demonstrated that *STAT1* knock-out mice (*STAT1^−/−^*) are susceptible to HSE [23] with an increased HSV-driven immune pathology in the cornea [24] and also in the brain stems [25]. *Unc93b1*-deficient mice have also been associated with HSE susceptibility despite their ability to control viral replication in the brain [26]. Altogether, these studies are consistent with the genetic etiologies of HSE reported in humans. Mice deleted for *Myd88*, which encodes for the adaptor protein of TLR7 and 9, were also highly susceptible to HSE [27]. In contrast to this finding, Honda et al. showed no increased susceptibility to HSE for *Myd88^−/−^* mice, whereas *Irf7^−/−^* had significantly increased mortality, correlating with reduced IFN-α level in the sera [28]. Like *Myd88*, *TLR9^−/−^* mice have been shown to be either more susceptible [29] or resistant [26] to HSE. The discrepancy in these studies may be due to different routes of infection or differences in viral strains. Nevertheless, these reports clearly demonstrate the key role of antiviral IFNs in mouse models of HSE. Several knock-out mice have also been used to study the immune control of HSE by NK, T or B cells. B cell-deficient mice were more susceptible to HSE [30] [31] whereas the role of T cells, in particular the role of CD8^+^ T cells, is still controversial [32] [33]. For example, athymic nude mice lacking T cells have been shown to be resistant to HSE similar to wild-type (WT) mice [34] while Metcalf et al. found them more susceptible to HSE [35]. Similar to that of T cells, the contribution of NK cells in HSE has been unclear [36] [37] [38]. Hence, the role of each of these immune cell populations remains elusive, as susceptibility to HSV-1 depends on both viral (viral strain, route of infection, and viral dose) and host factors (strain and age of the animal). These investigations have nonetheless paved the way for the study of the genetic etiology of HSE. However, no gene associated with HSE has thus far been identified by positional cloning in mice and host factors that contribute to susceptibility or resistance to this pathology also remain largely unknown.

N-Ethyl-N-Nitrosourea (ENU) mutagenesis constitutes an inherently unbiased, functional genomic strategy for the identification of genes, including those without known functions or biological precedents, regardless of their chromosomal location and expression pattern [39]. Treatment with this chemical mutagen introduces random point mutations in the mouse genome, with an average substitution rate of one nucleotide per megabase (Mb). In high throughput phenotypic screens, such mutations may lead to the identification of genes responsible for specific immune response defects and/or genes associated with diseases. Furthermore, positional cloning of mutant genes is now facilitated by recent progress in the field of genomics, such as exome sequencing and the availability of the mouse genome sequence. Thus, ENU mutagenesis has been used to successfully identify genes, proteins, and signaling pathways involved in a wide range of biological processes, including susceptibility to infection [40] [41], obesity [42], muscle development and function [43], cardiomyopathy [44], thrombocytopenia [45] and immunodeficiency [46]. This last report, describing a single-nucleotide mutation in the *Unc93b1* gene that abrogates signaling via TLR3, 7 and 9, has largely contributed in the discovery of the human UNC93B1 mutations [14].

In this study, we took advantage of the large scale mouse ENU mutagenesis platform developed in our laboratory to systematically search for novel host genes that directly impact infection by HSV-1 and the development of HSE. This led the identification of the *Ptprc* gene as a novel host factor associated with HSV-1 susceptibility.

## Results

### Description of the ENU-screening approach and identification of a HSV-1 susceptible mutant

**Figure 1:**
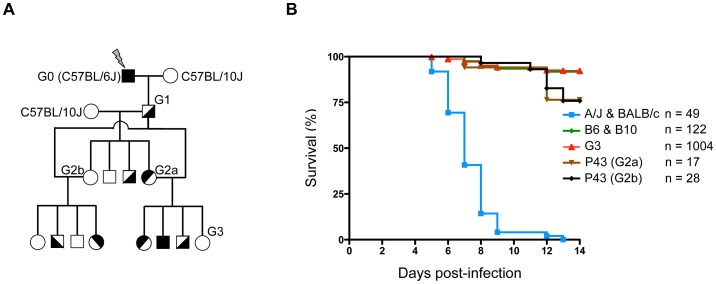
ENU-screening strategy. (**A**) Schematic representation of the breeding strategy used to produce G3 pedigrees. Details of the breeding procedure are described in the Materials and Methods (*ENU mutagenesis and breeding* section). (**B**) G3 pedigrees were screened for their susceptibility to HSV-1 infection. Mice were infected i.p. with 1×10^4^ pfu of HSV-1 strain 17 and survival was monitored for two weeks post-infection; susceptible A/J and BALB/c control mice are represented by a blue line, resistant C57BL/6J and C57BL/10J control mice are represented by a green line, all G3 mice by a red line and *P43* G3s derived from two G2 daughters (G2a and Gb) and one G1 male by brown and black lines. “n” indicates the number of infected mice for each group.

To identify novel host genes and fundamental pathways that directly impact HSV-1 infection and HSE pathology, ENU-mutagenized mice were screened for their susceptibility to HSV-1. Generation 0 (G0) C57BL/6J (B6) male mice were first treated with the mutagen ENU and then mated with C57BL/10J (B10, to facilitate subsequent genetic mapping) mice to produce G1 males, the founders of the colony which are heterozygous for ENU-induced mutations (Figure 1A). The G1 males were then backcrossed to B10 mice, to produce G2 animals. Two randomly chosen G2 daughters were backcrossed to their G1 father to produce G3 pedigrees, where homozygous ENU-mutations are expected to segregate in about 25% of animals. These G3 pedigrees were then infected i.p. with 1×10^4^ pfu of HSV-1 strain 17. This dose led to lethal encephalitis in susceptible A/J mice, whereas B6 mice remained unaffected (Figure S1). Following infection, the ENU-mutagenized mice were monitored for two weeks; the pheno-deviant offspring that exhibited clinical signs or succumbed to the infection were considered susceptible. Over 1,000 G3 mice, corresponding to sixty-nine G1 males, were screened for their susceptibility to HSV-1 infection. As shown in figure 1B, most A/J and BALB/c mice succumb uniformly between five and eight days post-infection (p.i.). In contrast, WT B6 and B10 mice, as well as ENU-mutagenized G3 mice, are susceptible in less than 10% of cases, which is largely under the expected frequency of susceptibility in pedigrees segregating a recessive mutation (25%). Sixty-nine G1 pedigrees were screened for their susceptibility to HSV-1 infection. Of these, a pedigree called *P43* appeared promising. Indeed, mating the G1 male with either of the two G2 daughters (G2a and G2b in Figure 1A) produced HSV-1 susceptible G3 offspring with over 20% of mortality upon infection (Figure 1B). These susceptible animals generally succumbed later than in A/J and BALB/c mice, between day 11 and 13 p.i. Moreover, no difference was observed between male and female offspring (data not shown).

### Susceptibility to HSV-1 infection is caused by a mutation in the *Ptprc* gene

**Figure 2:**
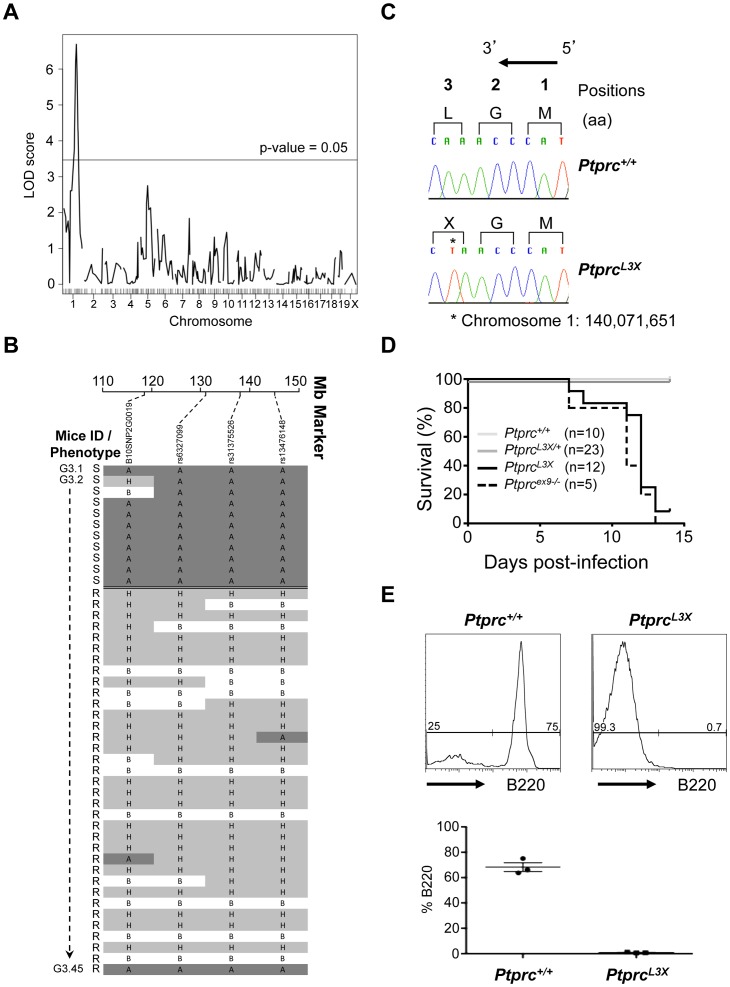
Identification and characterization of an HSV-1 susceptible ENU mutant. (**A and B**) Genome-wide linkage analysis was performed in 45 G3 mice from the *P43* pedigree (11 susceptible, 34 resistant) using polymorphic markers distinguishing the B6 and B10 genetic backgrounds. (**A**) Genome-wide linkage quantitative trait loci (qtl) analysis revealed a significant peak on chromosome 1 (LOD score = 6.7, calculated using survival as the phenotype). The horizontal line represents the threshold of significant LOD scores genome-wide, at a p-value = 0.05. (**B**) Haplotype analysis of the distal portion of chromosome 1 are shown for all G3 mice from the *P43* pedigree (A, dark gray shading = B6 homozygote; B, white = B10 homozygote; H, light gray = B6/B10 heterozygote; S = susceptible animal; R = resistant animal). (**C**) Genomic DNA from two G3 *P43* pedigrees (one resistant and one susceptible) was amplified by polymerase chain reaction and the star indicates the position of the *Ptprc^L3X^* mutation. DNA sequence electropherograms are shown for both resistant and susceptible mice and each of the four nucleotides were labeled with a unique fluorescent dye. Amino acid (aa) positions are displayed in bold; the black arrow indicates the direction of transcription. (**D**) *Ptprc^L3X^*, *Ptprc^L3X/+^*, *Ptprc^+/+^* and *Ptprc^ex9−/−^* mice were infected i.p. with 1×10^4^ pfu of strain 17 and monitored for survival. “n” indicates the number of infected mice for each group. (**E**) Blood from *Ptprc^+/+^* and *Ptprc^L3X^* mice was collected and PBMCs were isolated and stained for B220 (n = 3). The expression of B220 was quantified by FACS (upper panel) and represented as a percentage of total cells (lower panel).

To map the locus responsible for the HSV-1 susceptibility we carried out a genome-wide scan on 45 G3 mice, including 11 susceptible and 34 resistant animals. Linkage analysis identified a significant quantitative trait loci (QTL) signal on chromosome 1 (position 118.6 to 144.9 Mb) with a logarithm of odds (LOD) score of 6.7 (Figure 2A). The critical interval was associated with homozygous B6-derived alleles from the original mutated G0 male, except in the case of one animal that survived the infection (Figure 2B, see G3.45). In contrast, homozygosity for B10 alleles and B6/B10 heterozygosity correlated perfectly with resistance. These findings suggested that HSV-1 susceptibility in the *P43* pedigree is due to a recessive mutation located between 118.6 and 144.9 Mb on chromosome 1.

This 26 Mb interval on chromosome 1 contains more than 250 genes. Thus, to identify the ENU induced mutation involved in our HSV-1 susceptibility phenotype, whole exome sequencing, which consists of selectively capture and sequencing the coding regions of the genome, was performed on genomic DNA from two *P43*-derived G3 mutants. The generated sequences were then compared to the WT B6 reference sequence, filtered against public and in house databases, and only genetic variations supported by a minimum coverage of 10 sequence reads were considered (Table 1). This represented a total of 116 mutations, of which 88 were homozygous and 13 were common to both sequenced *P43* mutant mice. Two mutations were localized in the distal region of chromosome 1 (118.6–144.9 Mb) previously identified by linkage analysis. One of these two mutations was a non-synonymous mutation I543F in the *Kcnt2* gene, which encodes for a protein belonging to the voltage-gated potassium channel complex. The structure of Kcnt2 is probably not affected by the ENU-induced mutation since I and F residues are both hydrophobic, neutral and non-polar amino acid. However, the other mutation is likely to have a significant impact at the protein level: a unique A to T transversion in exon 1 (chromosome 1∶140,071,651) of the *Receptor-type tyrosine-protein phosphatase C* (*Ptprc*) gene, which generates the premature stop codon L3X (Figure 2C). The *Ptprc* gene is expressed on all nucleated hematopoietic cells and encodes for the multiple forms of the tyrosine phosphatase receptor CD45, including the isoform B220 present on B cells. CD45, when analyzed with the Gene Ontology (GO) database, is statistically enriched for “response defense to virus” and thus represents the best candidate gene to explain the HSV-1 susceptibility observed in this pedigree. First, the L3X mutation in *Ptprc* was confirmed by Sanger sequencing of all affected (n = 11) and unaffected individuals (n = 34) of the *P43* pedigree. As shown in Figure 2D, the susceptibility to HSV-1 infection was associated with *Ptprc^L3X^* genotype in more than 90% of cases (11/12), whereas HSV-1 resistance segregated with *Ptprc^L3X/+^* and *Ptprc^+/+^* littermates. Furthermore, previously described *Ptprc* null mice, that lacked expression of all endogenous CD45 isoforms due to a targeted deletion on exon 9 (*Ptprc^ex9−/−^*) [47], were susceptible as homozygous *Ptprc^L3X^* animals (Figure 2D). Most notably, *Ptprc^ex9−/−^* mice succumbed within the same time-frame than our ENU-induced mutant mice, providing independent evidence that *Ptprc^L3X^* mutation determines the vulnerability to HSV-1 observed in pedigree *P43*. FACS analysis demonstrated that about 70% of peripheral blood mononuclear cells (PBMCs) from *Ptprc^+/+^* mice were stained with antibody against B220, whereas no signal was observed in PBMCs from mutant *Ptprc^L3X^* littermates (Figure 2E). A similar absence of B220^+^ cell compartment was also observed in the spleen of *Ptprc^L3X^* mice (data not shown). These results indicated that *Ptprc^L3X^* is a null allele resulting in complete absence of protein. In the following sections, *Ptprc^L3X/+^* were used as controls since these heterozygous littermates phenocopy *Ptprc^+/+^* mice in their response against HSV-1.

**Table 1:** Exome sequencing analysis.

	DNA SAMPLE 1*	DNA SAMPLE 2*
Total mutations >10	46	70
SS/NS/DM	13/26/7	12/48/10
Homozygous mutations	38	50
Shared mutations	13	
Chr1: 118.6–144.9 Mb	2	
Damaging mutation	1	

* Whole exome sequencing of genomic DNA from two *P43* mutants was performed. All mutations (heterozygous and homozygous mutations) supported by a minimum coverage of 10 are presented (SS, synonymous mutation; NS, non-synonymous mutation; DM, damaging mutation).

### *Ptprc* L3X mutation determines susceptibility to HSV1, independent of the infectious route and viral strain

**Figure 3:**
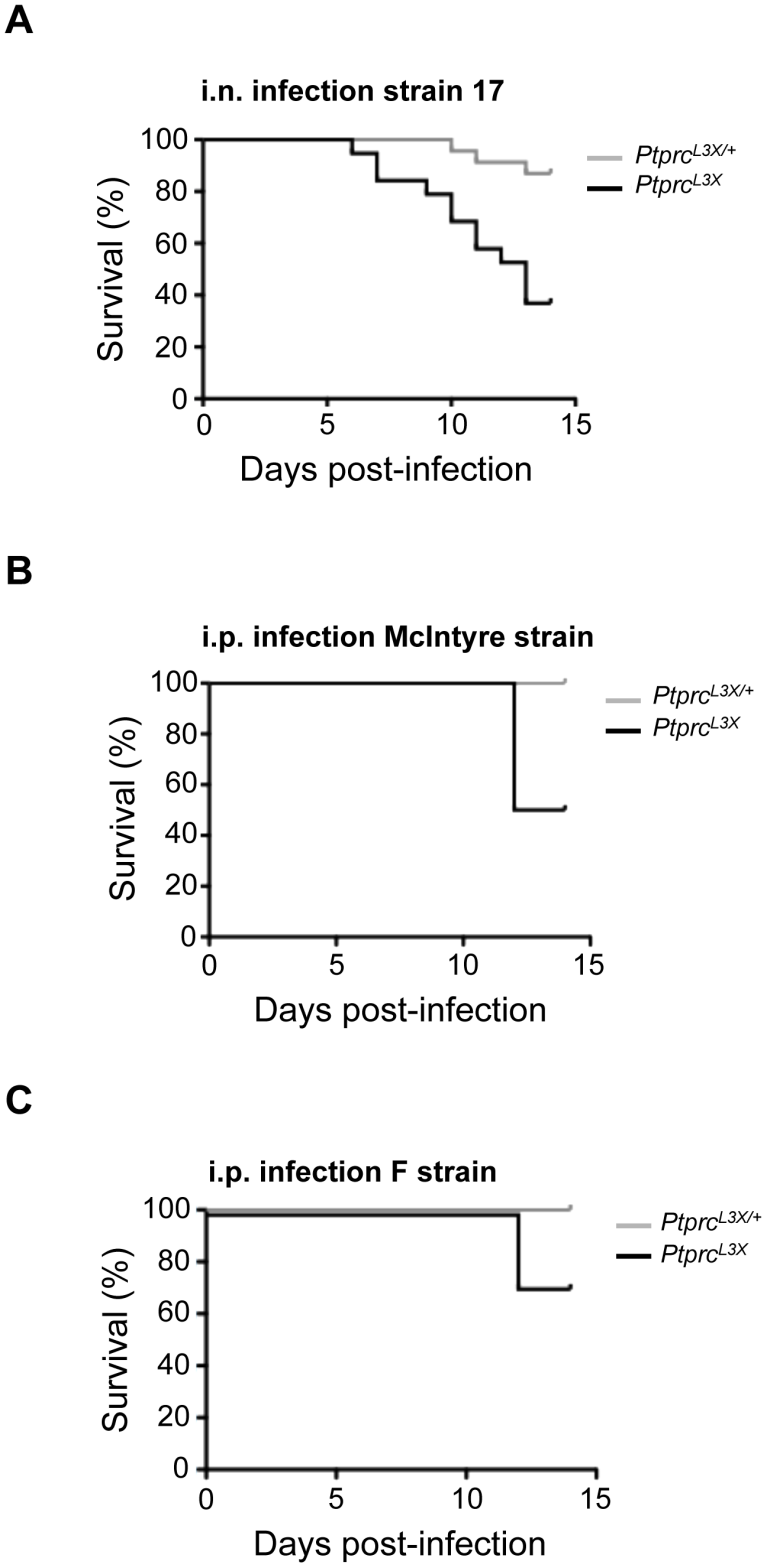
The HSV-1 susceptibility associated with the *Ptprc^L3X^* genotype is independent of the infection route and HSV-1 strain. (**A**) *Ptprc^L3X^* and heterozygous littermates were infected i.n. with 5×10^3^ pfu of HSV-1 and monitored for survival. (**B and C**) *Ptprc^L3X/+^* and *Ptprc^L3X^* mice were infected i.p. with 1×10^4^ pfu of either McIntyre (**B**) or F strain (**C**) and monitored for survival. n≥6 for each group and data represent at least two independent experiments. (**A, B and C**).

To determine a possible impact of the infection route on the survival phenotype, *Ptprc^L3X^* mice and heterozygous littermates were infected intranasally (i.n.) with 5×10^3^ pfu of HSV-1 strain 17. This dose led to lethal encephalitis in susceptible A/J mice by day 6–7 post-infection, whereas all B6 mice survived (Figure S2). In this infectious model, more than 60% of *Ptprc^L3X^* mice succumbed around the same time (by day 11–13 p.i.) as those infected i.p., while heterozygous littermates survived in more than 85% of cases (p-value = 0.003) (Figure 3B). Furthermore, i.p. infection with the McIntyre or F strains of HSV-1 also resulted in an increased susceptibility in *Ptprc^L3X^* mice only (Figure 3C and 3D, respectively), despite the fact that, relative to strain 17, the McIntyre and F strains appear less virulent in our experimental model (compare survival curves in Figure 2D, 3C and 3D). Thus, the association between our HSV-1 susceptibility phenotype and the *Ptprc^L3X^* genotype is independent of the infection route and HSV-1 strain.

### HSV-1 susceptibility is associated with profound CNS inflammation

**Figure 4:**
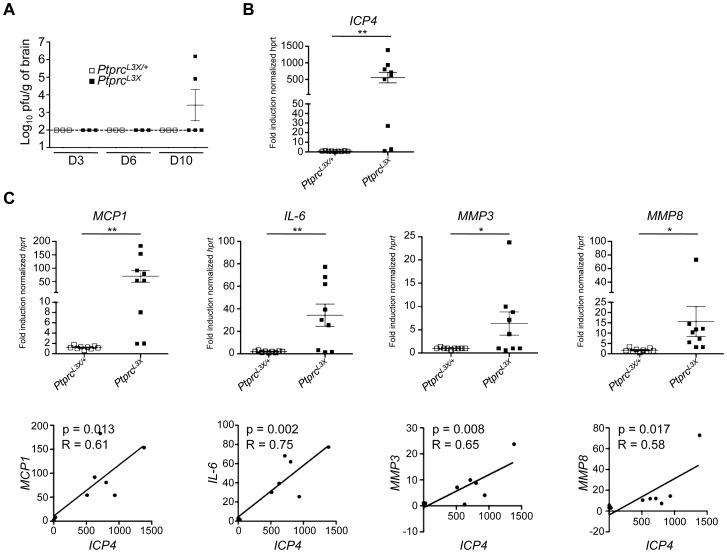
Expression of viral and inflammatory host genes in brain tissue collected from *Ptprc^L3X/+^* and *Ptprc^L3X^* infected mice. (**A**) *Ptprc^L3X/+^* and *Ptprc^L3X^* mice were infected i.p. with 1×10^4^ pfu of HSV-1. At the indicated day (D3, D6 and D10), total brains were harvested and mechanically homogenized for a plaque assay (n≥3). Viral titers are presented as pfu/gram of brain. The dotted line indicates the threshold of detection. (**B and C**) *Ptprc^L3X/+^* and *Ptprc^L3X^* mice were infected i.p. with 1×10^4^ pfu of HSV-1 (n = 9). Following infection, these mice were weighed two times daily. The brain stems of *Ptprc^L3X^* mice that had lost at least 15% of their pre-infection weight were harvested. *Ptprc^L3X/+^* mice were sacrificed and their brain stems were collected at days 7, 9, and 11 p.i. (n = 3 for each time point). The expression of the *ICP4* viral gene (**B**) and the indicated cellular genes (**C, upper panels**) was normalized to that of *hprt*. Data are presented as a fold increase relative to infected B6 samples. *, p-value (p)<0.05 and **, p<0.005. Correlations of expression levels were determined by comparing *ICP4* and the indicated cellular genes (**C, lower panels**).

To better characterize the pathogenesis in our mouse model, we followed the kinetics of viral replication in the brain and periphery (spleen and liver) following i.p. infection. At days 3, 6, and 10 p.i., the spleen and liver from both *Ptprc^L3X/+^* and *Ptprc^L3X^* mice had undetectable levels of virus (data not shown). In contrast, the brain tissue from two out of five *Ptprc^L3X^* mice showed significant viral titers at day 10 p.i. (Figure 4A), which is consistent with results presented in Figure 2D, where a large number of these mice succumb at days 11–13 p.i. Otherwise, the brains from all *Ptprc^L3X/+^* mice had undetectable viral titers, correlating with their survival. To confirm this result, we carried a second set of experiments where mice were weighed twice daily and their brain stems were harvested if the mice lost at least 15% of their pre-infection weight. The brain stems were chosen instead of whole brains because *STAT1^−/−^* mice, which are also susceptible to HSV-1, have been shown to exhibit a high viral titer and upregulate a large number of inflammatory markers in their brain stems following HSV-1 infection [25]. As a measure of virus load, we determined the expression of the *ICP4* viral gene in the brain stems collected from *Ptprc^L3X/+^* and *Ptprc^L3X^* mice using real time quantitative PCR (qPCR). In these conditions, *ICP4* expression in the brain stems from seven out of nine *Ptprc^L3X^* mice was increased by more than 20-fold relative to those from *Ptprc^L3X/+^* mice (Figure 4B). The same samples were used to determine the transcript level of MCP1, IL-6 and two matrix metalloproteases (MMP-3 and -8) that are highly expressed during HSE and can also cause the disruption of the blood-brain barrier (BBB) [48] [49] [50] [51] [52] [53] [54] [25]. The expression of these targeted transcripts was significantly increased in *Ptprc^L3X^* mice (Figure 4C, upper panels), accounting for hyper-inflammation and pathological damage in the brain of susceptible mice. Most importantly, there is a strong correlation between the presence of HSV-1 and the high expression of *MCP1*, *IL-6*, *MMP3*, and *MMP8* in the brain stems of *Ptprc^L3X^* mice (Figure 4C, lower panels). This experiment was also performed on *IL-1β*, *TNF-α*, *CCL-3*, *CCL-4* and *CCL-5* genes, and gave the same results (Figure S3A). However, the expression of the *β-actin* gene in *Ptprc^L3X^* infected mice was equivalent to that of *Ptprc^L3X/+^* infected mice (Figure S3B), suggesting that the increased expression seen in the brain stems of *Ptprc^L3X^* mice results from an overwhelming inflammatory response. Altogether, these findings demonstrate that the HSV-1 susceptibility observed within this pedigree is associated with profound CNS inflammation, suggesting BBB lesions, both caused by uncontrolled viral replication in the brain.

### *Ptprc* L3X mutation is associated with absence of CD3^+^ T cells, which is due to defects in early stages of thymocyte development

**Figure 5:**
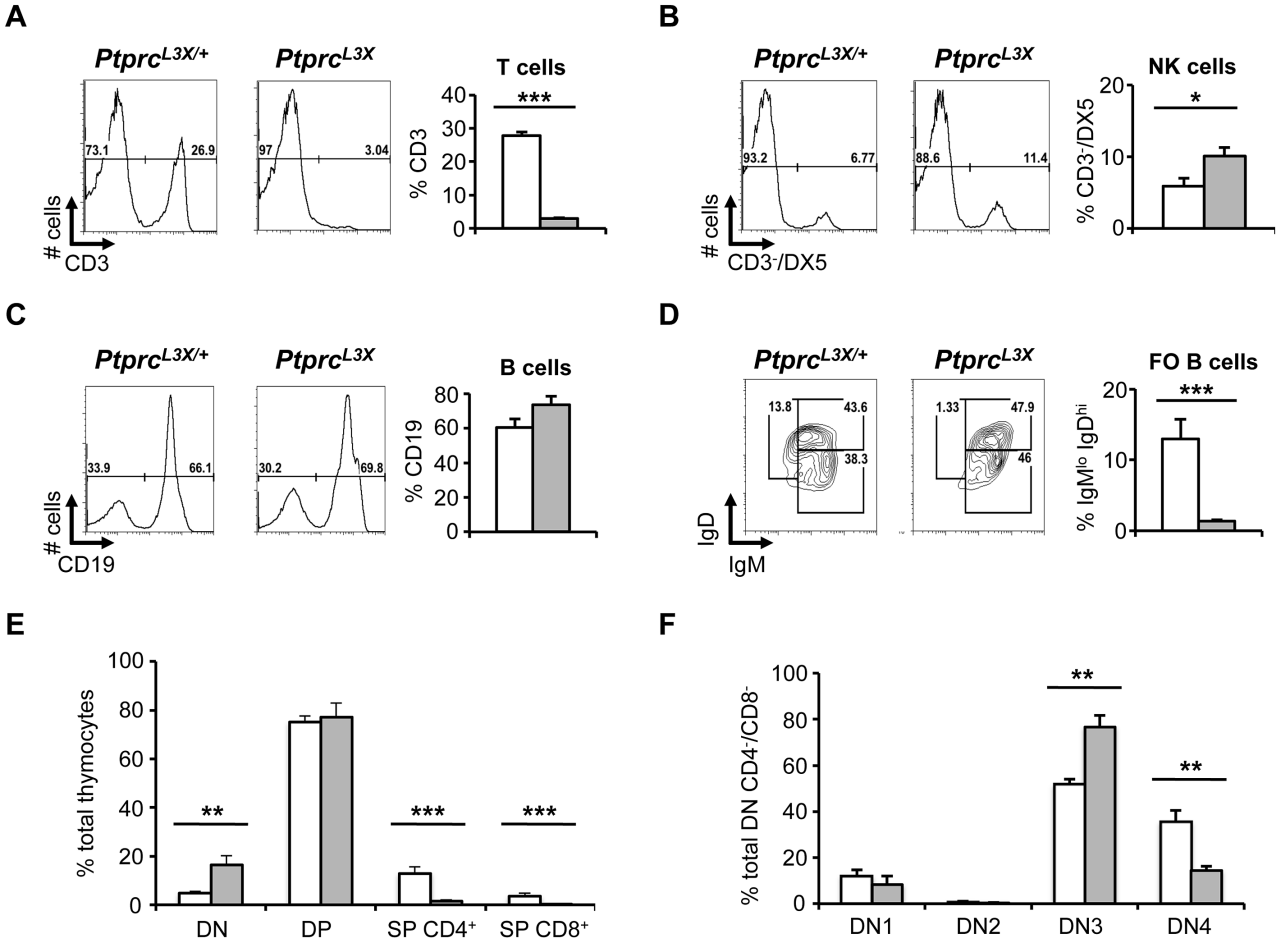
Immunological phenotyping. (**A, B, C and D**) The spleen of *Ptprc^L3X/+^* and *Ptprc^L3X^* mice was collected and specific immune cell populations were analyzed by FACS (n = 3). Isolated splenocytes were stained for CD3, DX5 and CD19 as well as IgD and IgM; their expressions were quantified by FACS and represented as a percentage of total cells. (**E**) Thymocytes isolated from *Ptprc^L3X/+^* and *Ptprc^L3X^* mice were stained for CD4 and CD8 and their expression were represented as a percentage of total cells. (**F**) Thymocytes were gated on CD4^−^CD8^−^ (double negative, DN); they were then analyzed based on their CD25 and CD44 expression profiles (DN1: CD25^−^CD44^+^; DN2: CD25^+^CD44^+^; DN3: CD25^+^CD44^−^; DN4: CD25^−^CD44^−^). CD25 and CD44 expression are presented as a percentage of total CD4^−^CD8^−^ double negative cells and are averaged over three animals. *, p<0.05; **, p<0.005 and ***, p<0.0005. *Ptprc^L3X/+^* and *Ptprc^L3X^* are shown in white and grey, respectively (**A, B, C, D, E and F**).

We have shown that HSV-1 susceptibility is associated with the presence of virus and profound inflammation in the CNS. This suggests that the immune response of susceptible mice could be affected, limiting their ability to control viral spread from the periphery into the CNS. To this end, the blood and spleen from *Ptprc^L3X^* and heterozygous littermates were collected and specific cell populations were examined by FACS. Of note, there was no significant difference in the total number of cells between *Ptprc^L3X^* and heterozygous littermates for these tissues (data not shown). Thus, FACS data are represented as a percentage of total cells. FACS analysis in *Ptprc^L3X^* mice revealed a lack of the CD3^+^ T cell compartment in the spleen, while heterozygotes showed normal percentages (Figure 5A). A similar absence of the CD3^+^ T cell compartment was also observed in the blood of *Ptprc^L3X^* mice (data not shown). Notably, DX5^+^/CD3^−^ NK cells from *Ptprc^L3X^* mice were increased two-fold relative to heterozygotes (Figure 5B), consistent with a study on mice defective for CD45 [55]. The percentage of CD19^+^ B cell and CD11c^+^/MHCII^+^ dendritic cell compartments from *Ptprc^L3X^* mice was unaffected (Figure 5C and data not shown). However, the IgD/IgM staining showed a reduced proportion of the mature follicular B CD19^+^/IgD^hi^/IgM^lo^ cells in mutants, suggesting defects in the final stage of B cell maturation (Figure 5D).

To gain insight into the absence of CD3^+^ T cells in the periphery, thymuses from *Ptprc^L3X^* and heterozygous littermates were collected and thymocytes were first stained for CD4 and CD8. As above, thymocytes from both *Ptprc^L3X^* and heterozygous littermates were present in equal numbers (data not shown). Consistent with results presented in Figures 5A, CD4^+^ and CD8^+^ single positive (SP) thymocytes were not present in *Ptprc^L3X^* mice (Figure 5E). However, CD4^−^CD8^−^ double negative cells (or DN) from *Ptprc^L3X^* mice were 4-fold higher relative to heterozygous littermates, suggesting a block at the DN stage. Then, the DN thymocytes were analyzed on the expression profiles of CD25 and CD44, two receptors expressed during T cell development. Thymocytes from *Ptprc^L3X^* mice displayed an accumulation of immature CD4^−^CD8^−^ DN3 cells (CD25^+^CD44^−^) and a reduced number of DN4 (CD25^−^CD44^−^) cells relative to heterozygous littermates (Figure 5F); these last results revealed a partial block at the β selection step of TCR-β rearrangement. These findings demonstrate that the *Ptprc* L3X mutation drastically affects the early stages of T cell development and could explain the HSV-1 susceptibility observed in the *P43* pedigree.

### Both CD4^+^ and CD8^+^ T cells are required for complete protection against HSV-1

**Figure 6:**
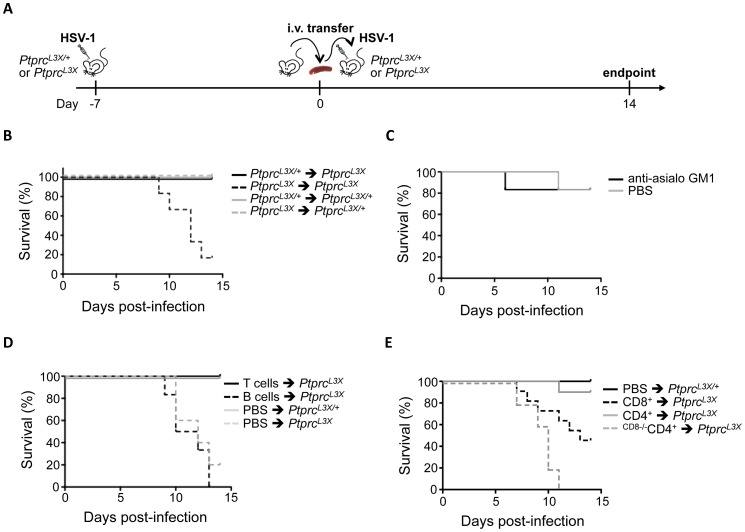
CD4^+^, without CD8^+^ T cells, are incapable to protect mice against lethal HSV-1 infection. (**A**) Schematic representation of the adoptive transfer strategy. Details of this procedure are described in the Materials and Methods (*Cell transfer experiments and NK depletion* section). (**B**) *Ptprc^L3X/+^* and *Ptprc^L3X^* mice received 2×10^7^ total splenocytes from either *Ptprc^L3X/+^* or *Ptprc^L3X^* mice. After two hours, these mice were infected i.p. with 1×10^4^ pfu of HSV-1 and their survival was monitored for two weeks (n = 6). (**C**) *Ptprc^L3X/+^* mice were treated with either anti-asialo GM1 antibody or PBS. After 24 hours, these mice were infected i.p. with 1×10^4^ pfu of HSV-1 and their survival was monitored for two weeks (n = 6). The injection of either anti-asialo GM1 antibody or PBS was performed every three days until the experimental endpoint. (**D**) *Ptprc^L3X^* mice received either 5×10^6^ T cells or 1.2×10^7^ B cells from *Ptprc^L3X/+^* mice. *Ptprc^L3X/+^* and *Ptprc^L3X^* mice that received only PBS respectively correspond to the positive and negative controls. After two hours, all mice were infected i.p. with 1×10^4^ pfu of HSV-1 and survival was monitored for two weeks (n≥6). (**E**) *Ptprc^L3X^* mice received either 2.5×10^6^ CD8^+^ or 2.5×10^6^ CD4^+^ T cells from either *Ptprc^L3X/+^* or *B6.H2-D^b^K^b^* knock-out (only for the CD4^+^ T cells transfer) mice, whose *H2-D^b^K^b^* make them depleted in CD8^+^ T cells (CD8^−/−^). *Ptprc^L3X/+^* mice that received only PBS correspond to the positive control. After two hours, all mice were infected i.p. with 1×10^4^ pfu of HSV-1 and survival was monitored for two weeks (n≥6). Data represent two independent experiments. (**B–E**).

We have shown that the L3X mutation in *Ptprc* leads to a lack of the CD3^+^ T cell compartment and affects B cell maturation. However, CD45 encoded by *Ptprc* has also been shown to play an important role in NK cell function, even if the phenotypic and functional consequences of CD45 deficiency are less severe than that of T cells [56]. Thus, through *in vivo* experiments, we attempted to establish which of the affected immune cell populations might be involved in the susceptibility to HSV-1 observed in *Ptprc^L3X^* mice. First, *Ptprc^L3X^* and heterozygous littermates were infected with HSV-1 before total splenocytes were harvested at day 7 p.i. This step is needed to prime the immune response. These splenocytes were then transferred into either *Ptprc^L3X^* or heterozygous littermates, before these mice were themselves infected with HSV-1 (Figure 6A). As expected, the transfer of total splenocytes from *Ptprc^L3X^* into *Ptprc^L3X^* mice did not rescue these mutant mice from lethal HSV-1 infection, while *Ptprc^L3X/+^* mice receiving splenocytes from either *Ptprc^L3X/+^* or *Ptprc^L3X^* mice survived (Figure 6B). On the other hand, the same cell transfer from *Ptprc^L3X/+^* into *Ptprc^L3X^* mice resulted in a complete HSV-1 protective effect. These results show that transfer of total spleen cells from *Ptprc^L3X/+^* mice to *Ptprc^L3X^* mice provide full protection against HSV1 infection. Next, we aimed to establish more precisely which immune cell population(s) is critical for the control of HSV-1 infection. In order to determine the possible role of NK cells in this protection, *Ptprc^L3X/+^* mice were treated with anti-asialo GM1 antibody, which is known to completely deplete NK cells [57] (Figure S4), and then infected with HSV-1. As shown in Figure 6C, the treatment with anti-asialo GM1 antibody had no impact on the survival of *Ptprc^L3X/+^* mice, suggesting that NK cells do not play a protective role against HSV-1. To distinguish the involvement of B cells from that of T cells in the protection against HSV-1, cells from *Ptprc^L3X/+^* mice were magnetically sorted and the individual sub-populations transferred into *Ptprc^L3X^* mice. While the transfer of B cells failed to rescue *Ptprc^L3X^* mice from lethal infection, the transfer of CD3^+^ T cells led to total protection against HSV-1 in all *Ptprc^L3X^* mice (Figure 6D). To distinguish the relative contribution of CD8^+^ from that of CD4^+^ T cells in the HSV-1 protective effect, we carried a third set of transfer experiments where *Ptprc^L3X^* mice were either injected with CD8^+^ or CD4^+^ T cells. The transfer of CD8^+^ T cells rescued about 50% of *Ptprc^L3X^* mice from lethal infection (Figure 6E). In contrast to CD8^+^ T cells, the transfer of CD4^+^ T cells led to a total protection against HSV-1 in almost all (9/10) *Ptprc^L3X^* mice (Figure 6E). Altogether, these data suggest that both the CD8^+^ and CD4^+^ T cells are involved in the HSV-1 protective effect, even if the CD4^+^ T cells appear to be more important than CD8^+^ T cells. However, it should be noted that a small proportion of CD8^+^ T cells (∼5%) were usually still present after the CD4^+^ T cell enrichment (data not shown). Thus, to demonstrate the strict dependence of CD8^+^ T cells in the CD4-mediated protection against HSV-1, we performed further transfer experiments where CD4^+^ T cells were purified from *B6.H2-D^b^K^b^* knock-out mice, whose *H2-D^b^K^b^* make them depleted in CD8^+^ T cells. In this condition, transfer of CD4^+^ T cells failed to rescue *Ptprc^L3X^* mice from lethal infection (Figure 6E). This last result demonstrates that CD4^+^, without CD8^+^ T cells, are incapable of establishing an efficient immune response against HSV-1. Moreover, *B6.H2-D^b^K^b^* knock-out mice were susceptible to lethal HSV-1 i.p. infection (p-value = 0.003) and succumbed around the same time (by day 11–13 p.i.) as *Ptprc^L3X^* mice infected i.p. (Figure S5), thus confirming the crucial role of the CD8^+^ T cells in protective immunity to HSV-1.

### CD4^+^ T cell-secreted IFN-γ mediates CD8^+^ T cell entry into the primary site of infection

**Figure 7:**
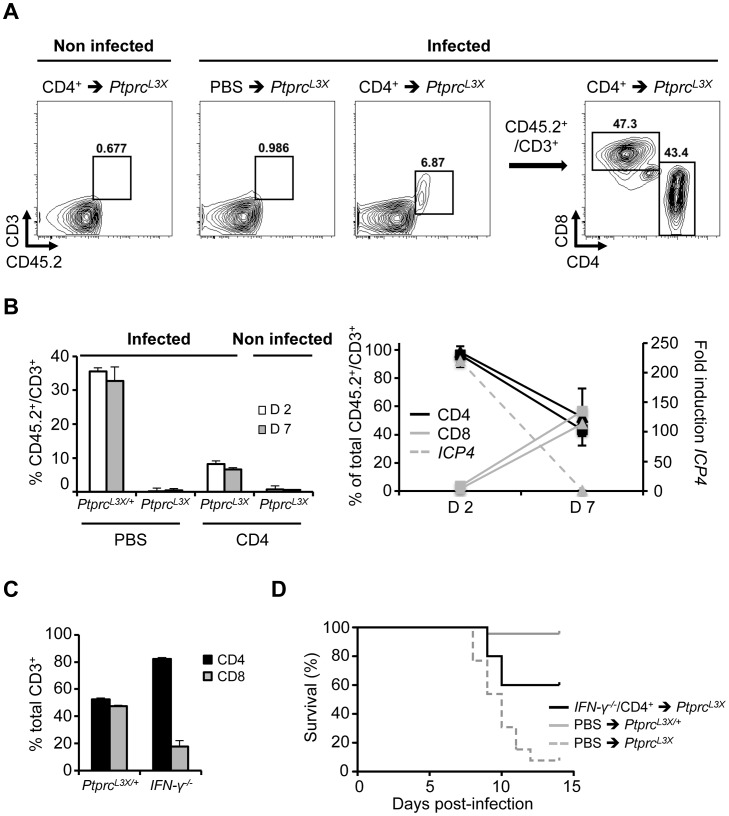
Important effect of IFN-γ production by the CD4^+^ T cells. (**A, B**) *Ptprc^L3X^* mice received 2.5×10^6^ CD4^+^ T cells from *Ptprc^L3X/+^* mice. *Ptprc^L3X/+^* and *Ptprc^L3X^* mice that received only PBS correspond to the positive and negative controls, respectively. After two hours and when indicated, mice were infected i.p. with 1×10^4^ pfu of HSV-1 and sacrificed at days 2 and 7 p.i (n = 3 for each group). At the indicated day, peritoneal cells were collected, stained for CD45.2, CD3, CD4 and CD8, quantified by FACS and gated on CD45.2^+^CD3^+^ (A, left panels, only shown for *Ptprc^L3X^* mice at day 7 p.i.); they were then analyzed based on their CD4 and CD8 expression profiles (**A, right panel,** only shown for *Ptprc^L3X^* infected mice that received CD4+ T cells from *Ptprc^L3X/+^* mice, day 7 p.i.). Proportion of CD45.2^+^CD3^+^ T cells was also represented as a percentage of total peritoneal cells (**B, left panel**), while expression of CD4 and CD8 were represented as a percentage of total CD45.2^+^CD3^+^ T cells (**B, right panel, left axis**). Triangles and squares correspond to *Ptprc^L3X/+^* and *Ptprc^L3X^* mice, respectively. The expression of the *ICP4* viral gene (**B, right panel, right axis**) was determined in total peritoneal cells collected from *Ptprc^L3X/+^* mice at days 2 and 7 p.i. *ICP4* expression was normalized to that of *hprt*. Data are presented as a fold increase relative to infected *Ptprc^L3X/+^* samples at day 7 p.i. (**C**) *Ptprc^L3X/+^* and *IFN-γ^−/−^* mice were infected i.p. with 1×10^4^ pfu of HSV-1 and sacrificed at day 7 p.i. Then, peritoneal cells were collected and were gated on CD45.2^+^CD3^+^. CD4 and CD8 expression are presented as a percentage of total CD45.2^+^CD3^+^ cells and are averaged over three animals. (**D**) *Ptprc^L3X^* mice received 2.5×10^6^ CD4^+^ T cells from *IFN-γ^−/−^* mice. *Ptprc^L3X/+^* and *Ptprc^L3X^* mice that received only PBS respectively correspond to the positive and negative controls. After two hours, all mice were infected i.p. with 1×10^4^ pfu of HSV-1 and survival was monitored for two weeks (n≥5).

To better characterize the contribution of the CD4^+^ T cells in CD8^+^ T cell function, we performed additional FACS experiments. *Ptprc^L3X^* mice were again transferred with CD4^+^ T cells purified from *Ptprc^L3X/+^* mice (same proportion of CD8^+^ T cells, as mentioned above, were still present after MACS separation, data not shown), and were then infected i.p. with HSV-1. Peritoneal cells, which in this infectious model are the first targets of HSV-1, were collected at days 2 and 7 p.i., gated on CD45.2^+^CD3^+^, and then analyzed for their CD4 and CD8 expressions. Here, we took advantage of the fact that *Ptprc^L3X^* mice do not express any CD45 receptors, including the isoform CD45.2 expressed by all leukocytes, to distinguish endogenous from transferred cells in the context of *Ptprc^L3X^* mice (Figure 7A). *Ptprc^L3X/+^* infected mice (PBS injected, positive control) showed about 35.5% of CD45.2^+^CD3^+^ cells in the peritoneal zone at day 2 and 32.7% at day 7 p.i., whereas *Ptprc^L3X^* infected mice (transferred with WT CD4^+^ T cells) exhibited 8.21% of CD45.2^+^CD3^+^ at day 2 and 6.6% at day 7 p.i. (Figure 7B, left panel). In contrast, *Ptprc^L3X^* infected mice (PBS injected, negative control) as well as those transferred with WT CD4^+^ T cells, but non-infected, did not show any T cells, suggesting that the recruitment of T cells to the peritoneal zone is infection-dependent. At day 2 p.i., we observed a high proportion of CD4^+^ T cells (>95% of total CD3^+^ T cells) in both *Ptprc^L3X/+^* (PBS injected) and *Ptprc^L3X^* (transferred with WT CD4^+^ T cells) infected mice. At day 7 p.i., however, the frequency of CD8^+^ T cells raised to 50% in equal proportions to CD4^+^ T cells, indicating CD8^+^ T cell recruitment to the site of infection (Figure 7B, right panel). Furthermore, this increased CD8^+^ T cell proportion perfectly correlated with the viral clearance (Figure 7B, right panel). Altogether, these results suggest that CD4^+^ help in mobilizing effector CD8^+^ T cells to the site of infection, which contributes to proper control of the dissemination of HSV-1 into the CNS. Recently, Nakanishi et al. demonstrated the role of CD4^+^ T cells, through their secretion of IFN-γ, in mobilizing effector CD8^+^ T cells to local HSV-2 infection [58]. Thus, we asked whether CD4^+^ T cells could mediate CD8^+^ T cell recruitment by the action of IFN-γ. To this end, IFN-γ knock-out mice (*IFN-γ^−/−^*) were infected, their peritoneal cells were collected at day 7 p.i. and the proportion of CD8^+^ relative to the CD4^+^ T cells was compared to infected *Ptprc^L3X/+^* mice. As shown in Figure 7C, *IFN-γ^−/−^* mice were significantly affected in their CD8^+^ T cell recruitment relative to the *Ptprc^L3X/+^* mice (p-value = 0.01 between *Ptprc^L3X/+^* and *IFN-γ^−/−^* CD8^+^ T cells). Furthermore, the CD4^+^ T cell transfer from infected *IFN-γ^−/−^* to *Ptprc^L3X^* mice did not rescue all of these mutant mice from lethal HSV-1 infection, suggesting that IFN-γ production by the CD4^+^ T cells is important, however, not indispensable (Figure 7D).

## Discussion

HSE is a lethal neurological disease resulting from infection with HSV-1. Previous studies have demonstrated a human genetic predisposition to HSE, involving the UNC93B-TLR3-type I IFN pathway in protective immunity to HSV-1. However, these gene mutations exhibit incomplete penetrance and represent only a minority of HSE cases, perhaps reflecting the effects of additional host genetics factors. Therefore, several groups have used mouse forward genetics to identify loci associated with susceptibility/resistance to HSE [21]. For example, Resistance to Herpes Simplex virus type 1 (Rhs1), a NK complex-linked locus present on chromosome 6, has been shown to be essential for the control of both acute and latent HSV-1 infection [59]. Another locus on the same chromosome, Herpes Resistance Locus (Hrl), has also been identified as a factor influencing HSV-1 infection and HSE pathology in mice [60]. More recently, a genome-wide linkage study has been performed in rats leading to the identification of the calcitonin receptor as a candidate gene for regulation of susceptibility to HSV-1 [61]. Thus, such *in vivo* approaches have illuminated natural variation in host components that contribute to CNS pathology in response to HSV-1 infection, notably by identifying loci associated with susceptibility/resistance to HSE; but no gene controlling resistance, however, has yet been identified by forward genetics.

In order to identify new genetic and cellular mechanisms involved in HSV-1 and HSE susceptibility, we have implemented the first genome-wide mutagenesis screen in an *in vivo* HSV-1 infectious model. Our high throughput approach allowed for the characterization of sixty-nine mouse pedigrees screened for their susceptibility to HSV-1, leading to the identification of our first susceptible mutant. Whole exome sequencing revealed two mutations, which are localized in the distal region of chromosome 1 (118.6–144.9 Mb) previously identified by linkage analysis. One of these two mutations is a non-synonymous mutation I543F in the *Kcnt2* gene, which encodes for a protein belonging to the voltage-gated potassium channel complex. Based on its high expression in the brain (http://biogps.gnf.org/), *Kcnt2* appeared promising, notably in the context of the CNS and viral encephalitis. Moreover, a gain-of-function mutation in the *Kcnt1* gene, a gene whose function is closely related to that of Kcnt2, has been recently associated with a childhood epileptic syndrome called malignant migrating partial seizures in infancy (MMPSI) [62]. However, and in contrast to the *Ptprc* L3X mutation, the *Kcnt2* I543F mutation is predicted to be benign when analyzed with the PolyPhen-2 database. Nevertheless, additional functional assays would be required to determine if *Kcnt2^I543F^* has any role in the infectious process. Indubitably, the large number of immunological mechanisms associated with *Ptprc* (http://amigo.geneontology.org/) as well as the HSV-1 susceptibility phenotype of *Ptprc^ex9−/−^* mice provide strong evidence that *Ptprc^L3X^* is causative of HSV-1 susceptibility observed in the *P43* pedigree.

*Ptprc^L3X^* presents distinctive features compared to other reported mutations within the *Ptprc* gene. First, the *Ptprc^L3X^* null mutation arose on a pure B6 chromosome. Other null mutations have been introduced in ES cells of 129 origin using a neomycine cassette targeting either exon 9 (*Ptprc^ex9−/−^*) or exon 12 (*Ptprc^ex12−/−^*) [47], [63]. Therefore, *ptrpc* mutations in mice *Ptprc^ex9−/−^* and *Ptprc^ex12−/−^* remain surrounded by 129-derived genetic material even after extensive backcrossing to B6. *Ptprc* is embedded in a polymorphic gene-rich chromosomal region that could influence immune, infectious or developmental phenotypes in knock-out mice. In comparable experiments, *Ptprc^L3X^* presents defects very similar to knock-out mice, including severely reduced numbers of T cells due to a defect in the transition from stage of development DN3 to DN4. *Ptprc^L3X^* is a null mutation and represents an excellent complement to two additional ENU-induced alleles, which were previously hypomorphic. *Ptprc^Loc^* and *Ptprc^Light^* were found in flow cytometric screens for recessive blood cell abnormalities. *Ptprc^Loc^* truncates the cytoplasmic domain of most CD45 isoforms but retains about 4% of wild-type CD45 expression [64]. *Ptprc^Light^* retains 15% of CD45 wild-type expression despite an F503S substitution in the transmembrane domain of the protein [65]. These allelic series in combination with *Ptprc^−/−^* mice has been used to demonstrate that the level of CD45 expression determines the dual role of CD45 in TCR signaling or T cell maturation. Now, the availability of *Ptprc^L3X^* mutant mice should allow a more accurate analysis of the full spectrum of CD45 functions.

In this study, we demonstrated that the HSV-1 susceptibility observed in our pedigree was independent of the infection route and was not viral strain-specific, two factors that are the leading causes of disparities observed in different mouse studies for HSV-1 [21]. We also report that this phenotype is associated with HSE pathogenesis characterized by a significant viral replication and a dramatic increase in the expression of proinflammatory mediators (MCP1 and IL-6) and enzymes (MMP3 and MMP8) in the brain stems. MCP1 and IL-6 have known implications in leukocyte recruitment and viral clearance in the brain. However, the modulation in their production is crucial to properly control the dissemination of viruses in the CNS without sustaining inflammation, which could result in significant neurological damage including the disruption of the blood-brain barrier (BBB). The BBB is a physical barrier between the peripheral circulation and the CNS [66] which contributes to prevent the exacerbation of leukocytes homing into the brain. In experimental mouse models of HSE and human cases, early findings demonstrate that this pathology leads to vascular alterations with disruption of the BBB [67] [68]. Other viral pathogens, such as human immunodeficiency virus 1 (HIV-1), human T-cell leukemia virus 1 (HTLV-1), lymphocytic choriomeningitis virus (LCMV), West Nile virus (WNV), rabies virus and mouse adenovirus type 1 (MAV-1), are also known to cause BBB disruption [54]. In most cases, the BBB damage is caused by indirect effects of viral replication in the CNS with the exception of HIV-1, where Tat, gp120 and Nef viral proteins have been directly involved. When highly expressed, MCP1 alters the actin cytoskeleton and localization of tight junction proteins in the brain endothelium, disrupting the BBB [69] [49]. Thus, MCP1 has been suggested to be a major determinant of brain damage after infection in mice [70], [71] and its high expression has been also correlated with HSE development [48]. Likewise, MMP3 and MMP8 are keys mediators of tight junction protein alterations, which degrade extracellular matrix proteins at the BBB leading to its disruption. Although IL-6 is protective against lethal HSV-1 ocular infection [51], elevated levels may be neurotoxic and also increase the permeability of the BBB [51] [50] [52] [53]. The high expression of *MCP1*, *IL-6*, *MMP3* and *MMP8* observed in the brain stems of *Ptprc^L3X^* mice are comparable to those reported by several studies evaluating the BBB integrity. While further investigations are still required to evaluate neurological damages, in particular the disruption of the BBB, in the brains of *Ptprc^L3X^* mice, these gene signatures nevertheless highlight the CNS inflammation during HSV-1 infection. Moreover, this demonstrates that our infectious experimental conditions represent a robust model for the identification of host factors that contribute to susceptibility or resistance to HSE. We were also able to establish a significant correlation between the presence of HSV-1 in the brain stems of mutant mice and the expression of *MCP1*, *IL-6*, *MMP3*, and *MMP8*. These last findings suggest that the post-HSV-1 infection mortality observed in this pedigree can be explained by a profound CNS inflammation caused by viral replication in the brain stem.

The first role demonstrated for CD45 was the regulation of T and B cell antigen receptor signaling via dephosphorylation of the Src family kinases (SFKs) Lck and Lyn, respectively (for a review, see [56]). In this study, we showed that the *Ptprc^L3X^* mutation drastically affects not only the early stages of thymocyte development but also the final stage of B cell maturation, consistent with previous studies that have used CD45 knock-out mice (for T cells, see [47], [63], [72], [73]; for B cells, see [74]–[76]). SFKs have been identified as its primary target, but CD45 is now known to also modulate the Janus kinases (Jaks) [77], as well as TLR signaling pathways [78] and NK receptor function [79]. CD45 functions have also been shown in mast cells, macrophages, and dendritic cells, as well as in leukocyte adhesion and migration [56]. While further experiments are still required to fully characterize our mutant in the several immune functions known for CD45, we have strong evidence that deficient T cell function largely contributes to HSV-1 susceptibility in mice. Indeed, we showed that the transfer of total splenocytes from *Ptprc^L3X/+^* into *Ptprc^L3X^* mice resulted in a complete HSV-1 protective effect; T cells were the only cell population to fully restore resistance to HSV-1 in the mutants, an effect that requires both the CD4^+^ and CD8^+^ T cells.

We also demonstrate that the presence of CD4^+^ T cells is a prerequisite for the CD8-mediated full protection against HSV-1. How CD4^+^ T cells provide help to CD8^+^ T cells – during priming, memory and/or mobilizing to the site of infection – remains a pending question, which will require further analysis. The critical role of the CD4^+^ T helper cells in the activation of the CD8^+^ T cells during the primary response to HSV-1, particularly in cytotoxic T lymphocyte priming, has been already demonstrated [80], [81]. In this study, we showed that the absence of IFN-γ leads to a partial but not absolute block in CD8^+^ T cells recruitment to the site of infection, consistent with the survival curve obtained from the CD4^+^ T cell transfer from *IFN-γ^−/−^* to *Ptprc^L3X^* mice. It should also be noted that the susceptibility of *IFN-γ^−/−^* mice to HSV-1 is still very controversial, as attested by different reports showing either susceptibility [82] or resistance [33], [83]. Using our infectious experimental conditions, *IFN-γ^−/−^* mice are resistant to HSV-1 as WT littermates (data not shown). Nevertheless, this finding is not necessarily in contradiction with our current data, but suggest that the only absence of IFN-γ is not sufficient to render resistant mice susceptible to lethal HSV-1 infection. Our findings also correlate with a study demonstrating the critical role of CD4^+^ Th1 cells after genital HSV-2 infection by timely recruitment of CD8^+^ T cells to the site of infection [58]. In this last publication, however, the critical role of CD4^+^ Th1 cells in CD8^+^ T cell mobilization to the site of infection for host survival was not established. Collectively, these results could also explain why the CD8^+^, without CD4^+^ T cells, only protected 50% of infected *Ptprc^L3X^* mice. Further investigations are needed to determine if other CD4^+^ T cell subset(s) (T regulatory cells, Th2, Th17…), and also which of the Tc1, Tc2 and Tc17 CD8^+^ T cell subset(s), contribute to rescue of *Ptprc^L3X^* mice from lethal HSV-1 infection. While we demonstrate here the critical role of both CD4^+^ Th1 and CD8^+^ T cells in protective immunity to HSV-1 using an unnatural inoculation route, we can reasonably propose that this adaptive immune mechanism also helps to control HSV-1 and maintain its latency in the CNS, as already been suggested by others [84], [85]. Thus, future studies to understand the key players in CD8^+^ T cell responses and their dependence on CD4^+^ T cell help should allow the generation of new prophylactic treatments against HSV-1 infection.

As mentioned above, CD45 plays a critical role in NK cell function and is required for protection from cytomegalovirus infection [79]. In contrast, CD45 deficiency protects mice from the lethal cardiomyopathy caused by Coxsackievirus B3 infection [77]. Likewise, others have demonstrated that intermediate levels, but not complete knock-out, of CD45 reduced apoptosis and protected mice from Ebola and *B. anthracis* infections [86], [87]. Altogether, while it is known that pathogens elicit divergent immune responses in mammals, our data are consistent with previous reports using phylogenetically distant pathogens whose control converges at CD45-mediated process. However, it remains to be determined if the varied effects of CD45 in the different infectious models reflect the multiple roles of CD45 in the establishment of an efficient immune response against pathogens, as it acts at different levels of both innate and adaptive immunity.

Since the main role of CD45 in lymphocytes is to promote cell activation, genetic alterations may lead to either lymphocyte hyper- or hypo-responsiveness. Thus, most studies performed in humans have focused on polymorphic variants that alter CD45 isoform expression, which are often associated with disease susceptibility [56], [88]. Absence of CD45 is routinely associated with severe combined immune deficiency (SCID). To this day, no mutation in *Ptprc* has been reported in a cohort of patients who suffer from HSE. Nevertheless, our findings do not necessary contradict current human data. One possibility is that *Ptprc* is not subject to specific genetic variation in human HSE, but remains mechanistically important for virus-host interactions. Alternatively, other alterations affecting T cell function could be associated with future human cases of HSE. For example, a strong correlation between decreasing CD4 count and increasing rates of HSV reactivation has been shown in individuals co-infected with HIV and HSV-2, suggesting that reactivation is linked to immunosuppression [89]. As mentioned in the introduction section, an autosomal recessive mutation (called 1757–1758delAG) in the *STAT1* gene was the first genetic etiology for HSE reported in HSV-1 seropositive patients [13]. This mutation has been associated with combined deficiencies in IFN-α/β, -λ and -γ signaling pathways since they rely on either STAT1/2 heterodimers (IFN-α/β and -λ) or STAT1 homodimers (IFN-γ). While the cellular phenotype of impaired STAT1 activation by IFN-α/β has been related to HSE susceptibility, we cannot exclude the possibility that an impaired response to IFN-γ in the infants also contributed to this disease, as documented for other viral susceptibilities [90], [91].

In sum, our screen for HSE identified a new CD45 null-allele causing susceptibility to HSV-1 infection. ENU mutagenesis is thus an effective tool for identifying potential causes of HSE. Characterization of susceptibility mutations in additional pedigrees should provide important information to rationalize alternative therapeutic strategies for a devastating, yet potentially preventable, disease.

## Materials and Methods

### Ethics statement

All mice were maintained under pathogen free conditions and handled according to the guidelines of the Canadian Council of Animal Care. The experimental protocol (Protocol number 4792) was approved by the McGill University ethics committee.

### Mice and viruses

C57BL/6J (B6), C57BL/10J (B10), A/J, BALB/c and *IFN-γ* knock-out mice were purchased from the Jackson laboratories (Bar Harbor, Maine, USA). ENU-mutagenized mice were bred in the animal facility of the Goodman Cancer Centre, McGill University. *Ptprc^ex9−/−^* and *B6.H2-D^b^K^b^* knock-out mice were kindly provided by Dr. Denis R. Alexander and Dr. Hidde L. Ploegh, respectively; *B6.H2-D^b^K^b^* knock-out mice possess targeted deletions in the *H2-D* and *H2-K* genes and are depleted in CD8^+^ T cells. HSV-1 strain 17 was originally from the laboratory of Dr. Subak-Sharpe whereas F and McIntyre strains were purchased from ATCC; these viral strains were amplified and titrated on Vero cells as previously described [92].

### ENU mutagenesis and breeding

Eight week-old G0 B6 male mice were mutagenized by i.p. injection of a fractionated dose of 3×90 mg/kg of ENU (Sigma) at weekly intervals. Efficiently mutagenized G0 males transiently lost and then regained fertility after 13 weeks. They were then out-crossed with B10 female mice to produce G1 offspring: these mice were F1 hybrids carrying one full set of mutagenized chromosomes and one full set of WT chromosomes. Individual G1 males were bred again to B10 females to generate G2 animals. Each G2 daughter inherits 50% of the B6 sequence variants in the G1. Two G2 females were then backcrossed to their G1 fathers to generate G3 animals, which were screened for their susceptibility to HSV-1 strain 17 infection.

### Mice infection and virus load

Mice 7 weeks of age or older were each infected intraperitonealy (i.p.) with 1×10^4^ plaque forming units (pfu) of HSV-1. When indicated, 4 week-old mice were each infected intranasally (i.n.) with 5×10^3^ pfu under anesthesia (5 mg/ml ketamine and 15 mg/ml xylazine). In this inoculation model, 20 µl of virus suspension was dripped directly into the nasal cavity in both nostrils of the mice. Infected mice were monitored 1–2 times daily over a 2-week period for survival. Mice that succumbed to infection within 14 days post-infection (p.i.) were considered susceptible to HSV-1. A/J and BALB/c mice were used as susceptible controls, while B6 and B10 mice were used as resistant controls. Organs of interest were collected from mice and then homogenized before being serially diluted 10-fold in non-supplemented DMEM and plated on Vero cells for plaque forming assays as previously described [92].

### DNA extraction and genetic mapping

Genomic DNA was isolated from tail biopsy by a standard phenol/chloroform extraction, as previously described [93]. Genome scanning was performed at the McGill University and Genome Quebec Innovation Centre (Montreal, Québec, Canada). DNA samples from 11 resistant and 34 susceptible G3 mice from the *P43* pedigree were analyzed with a panel of 255 B6/B10 polymorphic markers (SNPs) distributed across the genome [94] using massArray platform from Sequenom. Mapping data were analyzed using the R/qtl software, version 2.12.2. The binary model was used and LOD scores were calculated using survival as the phenotype.

### Statistical and bioinformatics analyses

Statistical analyses were conducted with the program R; linkage was performed with the package “R/qtl,” version R 2.12.2. The scanone function of the R/qtl library was used to perform maximum likelihood interval mapping (EM) of the phenotype on genetic markers. Significance values were evaluated with 10,000 permutations. The logarithm of the odds (LOD) support interval of QTL peaks was calculated using a 1.5 LOD drop by R/qtl. p-values are a result of two-tailed t-tests and R square (R) values are a result of linear regression tests.

All graphs represent the mean, and include error bars of the standard deviation.

### Whole-exome sequencing

Genomic DNA samples from two affected individuals were processed at the Centre National de Génotypage (Evry, France). Exome capture was performed using a SureSelect Mouse All Exon kit (Agilent Technologies, USA) and parallel sequencing on an Illumina HiSeq 2000 (100-bp paired end reads). This generated over 8 Gb of sequence. Reads were aligned to mouse genome assembly July 2007 (NCBI37/mm9) with Burrows-Wheeler Alignment (BWA) tool [95] and coverage was assessed with BEDTools, showing an average of 58.9 reads covering each base of the consensus coding sequence genes for the mouse genome [96]. Single nucleotide variants and short insertions and deletions (indels) were called using samtools pileup and varFilter [97] with the base alignment quality adjustment disabled, and were then quality filtered to require at least 20% of reads supporting the variant call. Variants were annotated using both Annovar [98] and custom scripts to identify whether they affected protein coding sequence, and whether they had previously been seen in mouse dbSNP128 or in any of 2 mouse exomes sequenced in parallel. In order to detect splice site mutations, the threshold of detection was increased to 6 bps instead of the standard 2 bps flanking exons.

### Quantitative PCR in brain tissue

Brain stems were harvested from sacrificed mice and immediately homogenized in 2 ml of trizol (Invitrogen). Total RNA was extracted using RNeasy columns (QIAGEN) and transcribed into cDNA using M-MLV with random hexamers (Invitrogen), according to the manufacturer's instructions. qPCR was performed using Platinum SYBR Green SuperMix-UDG (Invitrogen) together with 50 ng of reverse transcribed total RNA and experimental or control primers. Experimental primers targeting the *MMP3* and *MMP8* genes were designed to span exon junctions using primer3plus. For *MMP3*, the sequences used were 5′TGGAGATGCTCACTTTGACG3′ and 5′GCCTTGGCTGAGTGGTAGAG3′, while 5′TTTGATGGACCCAATGGAAT3′ and 5′GAGCAGCCACGAGAAATAGG3′ were used to quantify *MMP8* expression, 5′CACCACTGCCCTTGCTGTTCT3′ and 5′ ACACCTGGCTGGGAGCAAAG3′ for *CCL-3*, 5′TCTGCGTGTCTGCCCTCTCTC3′ and 5′GGCTTGGAGCAAAGACTGCTG3′ for *CCL-4*, 5′GATCTCTGCAGCTGCCCTCAC3′ and 5′CACACACTTGGCGGTTCCTTC3′ for *CCL-5*, 5′CAGGCAGGCAGTATCACTCA3′ and 5′AGGTGCTCATGTCCTCATCC3′ for *IL-1β*, 5′AGGTCATCACTATTGGCAACG3′ and 5′ATCTCCTTCTGCATCCTGTCA3′ for *β-actin*. The primers used to determine the expression of *MCP1*, *IL-6*, *TNF-α* and the *ICP4* viral gene have been previously described ([99] and [100], respectively). Target transcripts were normalized to the control housekeeping gene *hprt*. Reactions were performed in duplicate using the PTC200 Thermal Cycler with Chromo4 Continuous Fluorescence Detector (MJ Research). Expression was analyzed using Opticon Monitor 3 software (MJ Research). Relative mRNA expression levels were analyzed using the infected B6 samples as the reference group and calculated by subtracting the mean ΔCT of infected B6 samples from the ΔCT of the infected samples (ΔΔCT). The amount of target mRNA, normalized to the endogenous reference, was calculated as 2^−Δ(ΔCT)^.

### Immunophenotyping

Cells were isolated from spleen, blood or thymus as previously described [101]. Peritoneal cells were collected by lavage with a PBS-EDTA 10 mM solution: 3 ml of cold PBS was injected i.p. into the peritoneum, and the peritoneal area was vigorously massaged before the lavage fluid was withdrawn with a 3 ml-syringe. For each tissue, a minimum of 1×10^6^ cells were stained for 20 minutes at 4°C in the dark with the following antibodies (all from eBioscience): PerCP-Cy5.5 anti-CD19 (1D3), PerCP-Cy5.5 anti-CD3e (145-2C11), PE anti-DX5 (DX5), APC anti-B220 (RA3-6B2), FITC anti-CD8a (53-6.7), PE anti-CD4 (L3T4), PerCP-Cy5.5 anti-CD25 (PC61.5), APC anti-CD44 (IM7), PerCP-Cy5.5 anti CD45.2 (104), efluo-450 anti-CD8a (53-6.7), APC Cy7 anti-CD3e (17A2) and efluo-506 fixable viability dye. Live cells were gated using the forward scatter (FSC)-area versus viability plot, and then analyzed by FACS. Data analysis was performed using the FlowJo 9.3.1 software.

### Cell transfer experiments and NK depletion

Mice 7 weeks of age or older were each infected i.p. with 1×10^4^ pfu of HSV-1. One week later, spleens were collected and splenocytes were prepared using the aforementioned protocol (see Materials and Methods, *Immunophenotyping*). When indicated, 2×10^7^ total splenocytes were transferred intravenously (i.v.) into mice. Otherwise, CD19^+^ B cells, CD3^+^, CD8^+^ or CD4^+^ T cells were purified from total splenocytes using magnetic cell sorting (MACS; Miltenyi) according to the manufacturer's instructions. Mice were then injected i.v. with either 12×10^6^ CD19^+^ B cells, 5×10^6^ CD3^+^ T cells, 2.5×10^6^ CD8^+^ T cells or 2.5×10^6^ CD4^+^ T cells. After two hours, reconstituted mice were each infected i.p. with 1×10^4^ pfu of HSV-1 and were then monitored for survival. For NK cell depletion, mice 7 weeks of age or older were injected i.v. with 35 ul of anti-asialo GM1 antibody (Wako). After 24 hours, mice were each infected i.p. with 1×10^4^ pfu of HSV-1. To maintain NK cell depletion, the mice were treated with anti-asialo GM1 antibody every three days until the experimental endpoint. Throughout mice were monitored for survival.

## Supporting Information

Figure S1 **Pilot infection of the i.p. inoculation model.** WT A/J and C57BL/6J mice were infected i.p. with either 5×10^3^ (**A**), 1×10^4^ (**B**) or 1×10^5^ (**C**) pfu of HSV-1 strain 17. Survival was monitored for two weeks and all surviving mice were sacrificed at day 14 p.i. (experimental endpoint). n≥8 for each group.
(PDF)

Figure S2 **Pilot infection of the i.n. inoculation model.** Wild type A/J and C57BL/6J mice were infected i.n. with either 1×10^3^ (**A**), 5×10^3^ (**B**) or 5×10^4^ (**C**) pfu of HSV-1 strain 17. Survival was monitored for two weeks and all surviving mice were sacrificed at day 14 p.i. (experimental endpoint). n≥8 for each group.
(PDF)

Figure S3 **Expression of inflammatory molecules in the brain stems of** ***Ptprc^L3X/+^*** **and** ***Ptprc^L3X^*** **infected mice.** *Ptprc^L3X/+^* and *Ptprc^L3X^* mice were infected i.p. with 1×10^4^ pfu of HSV-1 (n = 9). Following infection, these mice were weighed two times daily. The brain stems of *Ptprc^L3X^* mice that had lost at least 15% of their pre-infection weight were harvested. *Ptprc^L3X/+^* mice were sacrificed and their brain stems were collected at days 7, 9, and 11 p.i. (n = 3 for each time point). The expression of the indicated cellular genes (**A, upper panels and B, left panel**) was normalized to that of *hprt*. Data are presented as a fold increase relative to infected B6 samples. *, p-value (p)<0.05, **, p<0.005 and “ns” for non significant. Correlations of expression levels were determined by comparing *ICP4* and the indicated cellular genes (**A, lower panels and B, right panel**).
(PDF)

Figure S4 **Depletion of NK cells by treatment of anti-asialo GM1 antibody.** (**A**) *Ptprc^L3X/+^* and *Ptprc^L3X^* mice were treated with either anti-asialo GM1 antibody or PBS. After 24 hours, these mice were infected i.p. with 1×10^4^ pfu of HSV-1 and were sacrificed 24 h later. The spleen and blood of *Ptprc^L3X/+^* and *Ptprc^L3X^* mice were collected (n = 3). Isolated cells were stained for CD3 and DX5; their expressions were quantified by FACS and represented as a percentage of total cells (the blood and spleen are shown in white and grey, respectively). (**B, C and D**) *Ptprc^L3X/+^* and *Ptprc^L3X^* mice were treated with either anti-asialo GM1 antibody or PBS. After 24 hours, these mice were infected i.p. with 1×10^4^ pfu of HSV-1 and their survival was monitored for two weeks (**B**, n≥3). The injection of either anti-asialo GM1 antibody or PBS was performed every three days until the experimental endpoint. At day 8 p.i. (**C**) the blood of both *Ptprc^L3X/+^* and *Ptprc^L3X^* mice were collected by cheek bleed, PBMC were isolated, stained for CD3 and DX5; their expressions were quantified by FACS and represented as a percentage of total cells. At day 14 p.i. (**D**, experimental endpoint), *Ptprc^L3X/+^* mice were sacrificed and their blood and spleen were collected. Isolated cells were stained for CD3 and DX5; their expressions were quantified by FACS and represented as a percentage of total cells (the blood and spleen are shown in white and grey, respectively). “ND” means non-determined.
(PDF)

Figure S5 **Susceptibility of** ***B6.H2-D^b^K^b^*** **knock-out mice to lethal HSV-1 i.p. infection.** *B6.H2-D^b^K^b^* knock-out mice and WT littermates were infected i.p. with 1×10^4^ pfu of HSV-1 strain 17. Survival was monitored for two weeks and all surviving mice were sacrificed at day 14 p.i. (experimental endpoint). Data represent two independent experiments, n≥12 for each group.
(PDF)
