# Supplementary figures and images for: Genome-Wide Mouse Mutagenesis Reveals CD45-Mediated T Cell Function as Critical in Protective Immunity to HSV-1
Source: PLoS Pathog. 2013 Sep 12;9(9):e1003637. doi: 10.1371/journal.ppat.1003637 (PMC3771889; doi:10.1371/journal.ppat.1003637)

**A**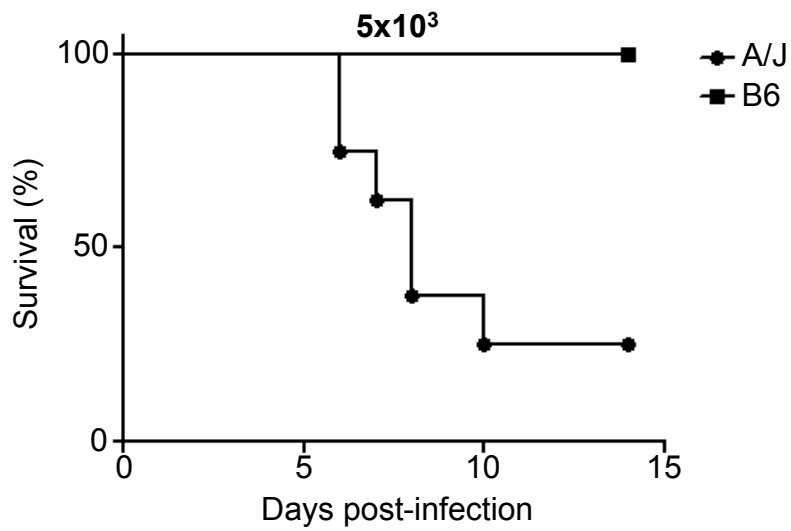**B**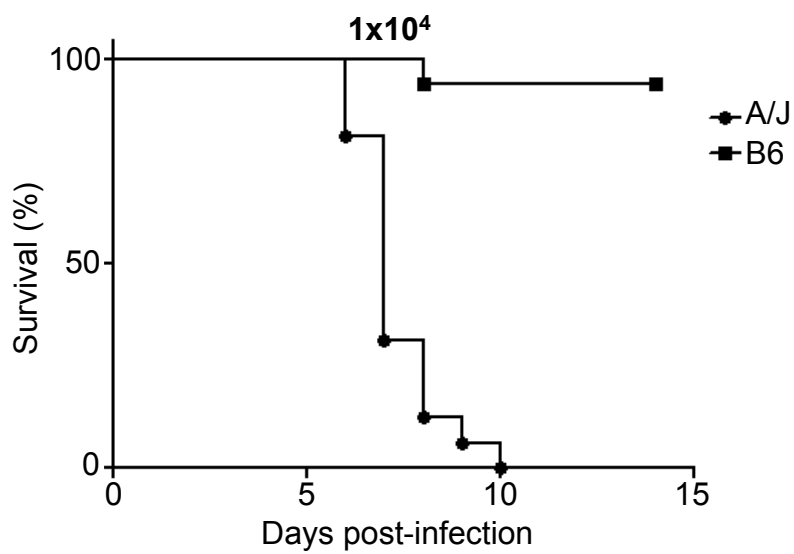**C**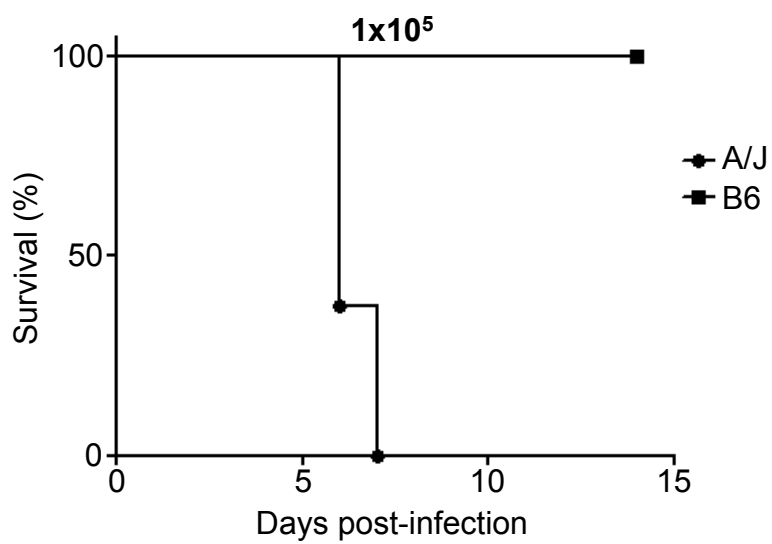

Supplement: Figure S1 — Pilot infection of the i.p. inoculation model. WT A/J and C57BL/6J mice were infected i.p. with either 5×10^3^ (A), 1×10^4^ (B) or 1×10^5^ (C) pfu of HSV-1 strain 17. Survival was monitored for two weeks and all surviving mice were sacrificed at day 14 p.i. (experimental endpoint). n≥8 for each group. (PDF) [file ppat.1003637.s001.pdf]

**A**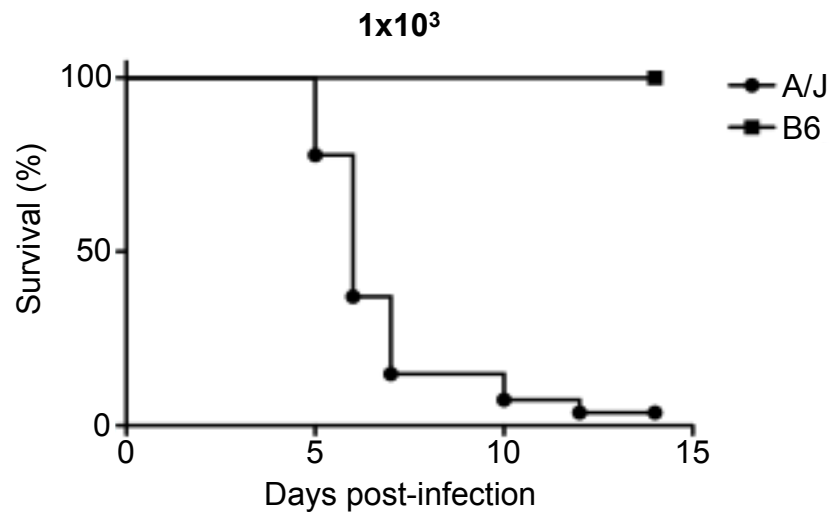**B**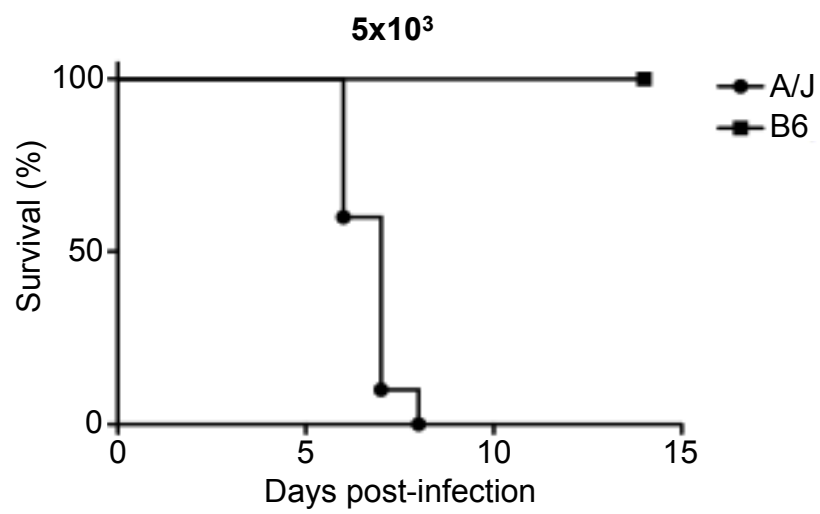**C**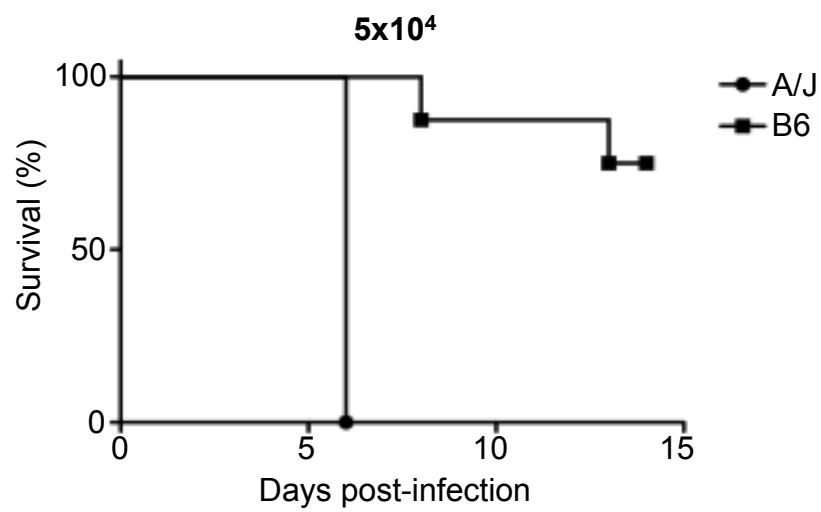

Supplement: Figure S2 — Pilot infection of the i.n. inoculation model. Wild type A/J and C57BL/6J mice were infected i.n. with either 1×10^3^ (A), 5×10^3^ (B) or 5×10^4^ (C) pfu of HSV-1 strain 17. Survival was monitored for two weeks and all surviving mice were sacrificed at day 14 p.i. (experimental endpoint). n≥8 for each group. (PDF) [file ppat.1003637.s002.pdf]

**A**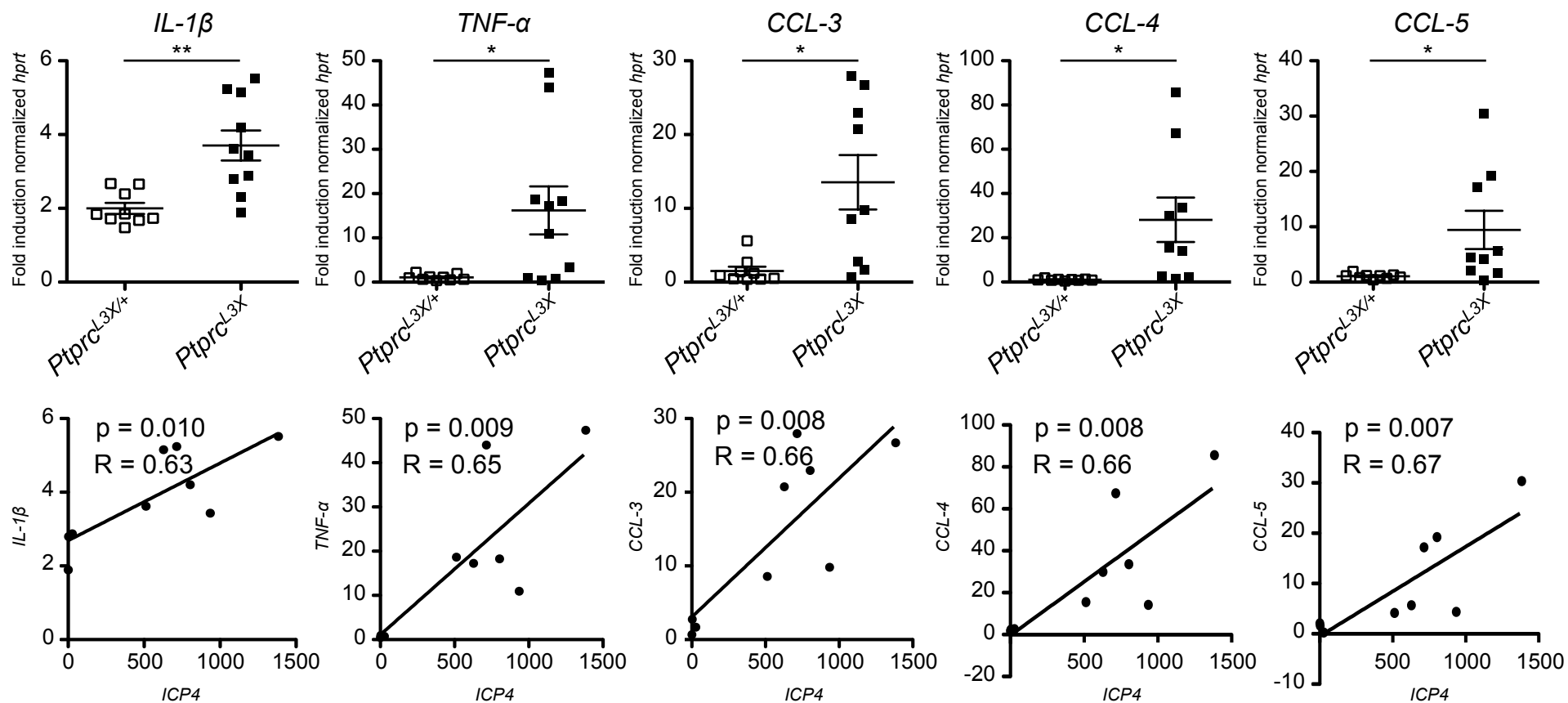**B**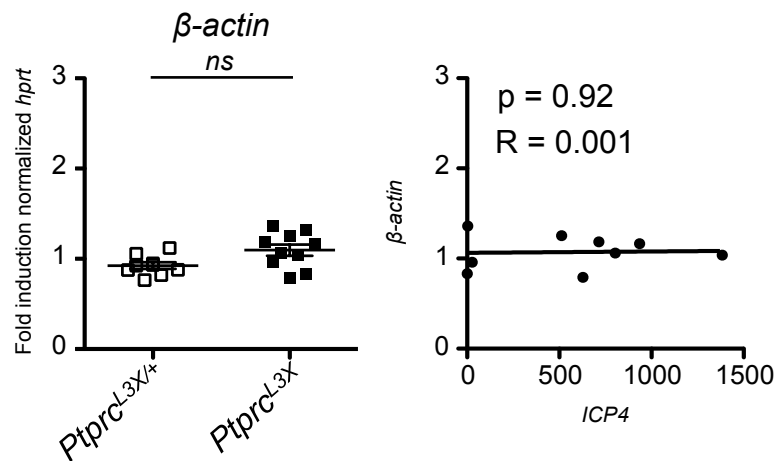

Supplement: Figure S3 — Expression of inflammatory molecules in the brain stems of PtprcL3X/+ and PtprcL3X infected mice. PtprcL3X/+ and PtprcL3X mice were infected i.p. with 1×10^4^ pfu of HSV-1 (n = 9). Following infection, these mice were weighed two times daily. The brain stems of PtprcL3X mice that had lost at least 15% of their pre-infection weight were harvested. PtprcL3X/+ mice were sacrificed and their brain stems were collected at days 7, 9, and 11 p.i. (n = 3 for each time point). The expression of the indicated cellular genes (A, upper panels and B, left panel) was normalized to that of hprt. Data are presented as a fold increase relative to infected B6 samples. *, p-value (p)<0.05, **, p<0.005 and “ns” for non significant. Correlations of expression levels were determined by comparing ICP4 and the indicated cellular genes (A, lower panels and B, right panel). (PDF) [file ppat.1003637.s003.pdf]

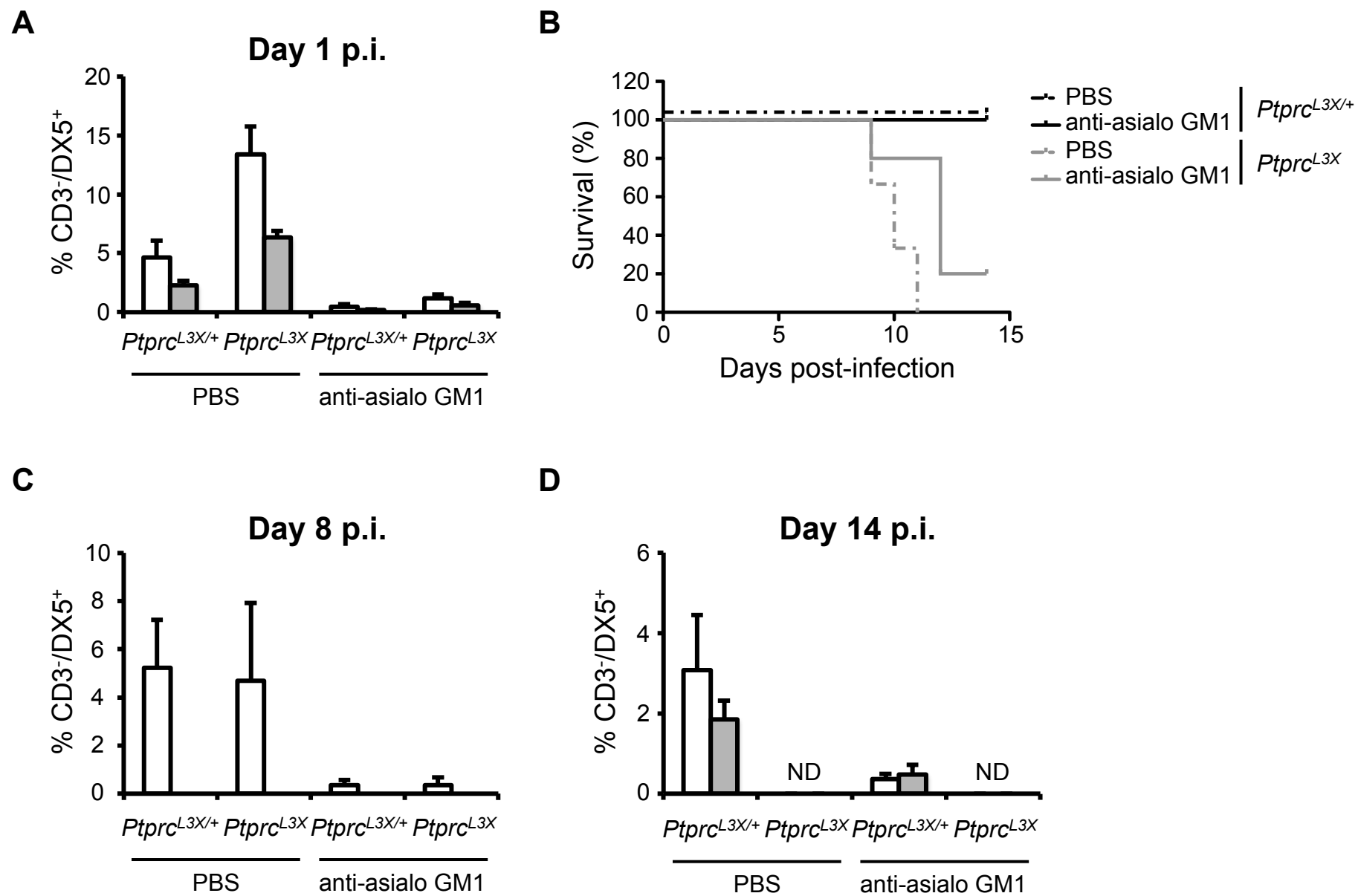

Supplement: Figure S4 — Depletion of NK cells by treatment of anti-asialo GM1 antibody. (A) PtprcL3X/+ and PtprcL3X mice were treated with either anti-asialo GM1 antibody or PBS. After 24 hours, these mice were infected i.p. with 1×10^4^ pfu of HSV-1 and were sacrificed 24 h later. The spleen and blood of PtprcL3X/+ and PtprcL3X mice were collected (n = 3). Isolated cells were stained for CD3 and DX5; their expressions were quantified by FACS and represented as a percentage of total cells (the blood and spleen are shown in white and grey, respectively). (B, C and D) PtprcL3X/+ and PtprcL3X mice were treated with either anti-asialo GM1 antibody or PBS. After 24 hours, these mice were infected i.p. with 1×10^4^ pfu of HSV-1 and their survival was monitored for two weeks (B, n≥3). The injection of either anti-asialo GM1 antibody or PBS was performed every three days until the experimental endpoint. At day 8 p.i. (C) the blood of both PtprcL3X/+ and PtprcL3X mice were collected by cheek bleed, PBMC were isolated, stained for CD3 and DX5; their expressions were quantified by FACS and represented as a percentage of total cells. At day 14 p.i. (D, experimental endpoint), PtprcL3X/+ mice were sacrificed and their blood and spleen were collected. Isolated cells were stained for CD3 and DX5; their expressions were quantified by FACS and represented as a percentage of total cells (the blood and spleen are shown in white and grey, respectively). “ND” means non-determined. (PDF) [file ppat.1003637.s004.pdf]

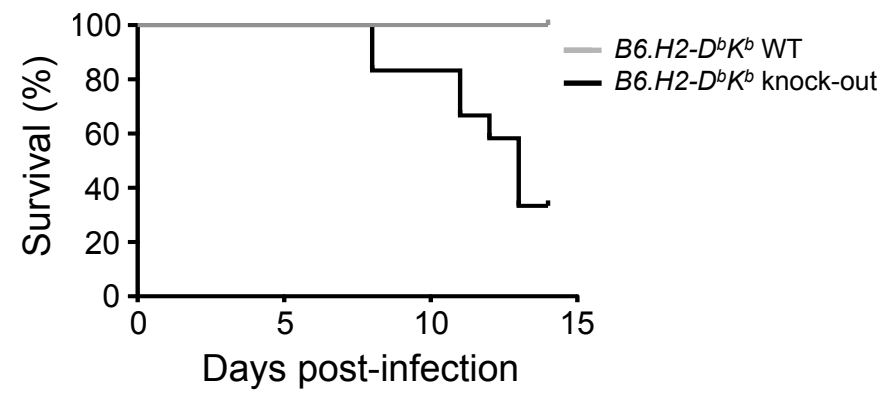

Supplement: Figure S5 — Susceptibility of B6.H2-DbKb knock-out mice to lethal HSV-1 i.p. infection. B6.H2-DbKb knock-out mice and WT littermates were infected i.p. with 1×10^4^ pfu of HSV-1 strain 17. Survival was monitored for two weeks and all surviving mice were sacrificed at day 14 p.i. (experimental endpoint). Data represent two independent experiments, n≥12 for each group. (PDF) [file ppat.1003637.s005.pdf]
